# Multicomponent mechanochemical synthesis

**DOI:** 10.1039/c7sc05370c

**Published:** 2018-01-29

**Authors:** Marco Leonardi, Mercedes Villacampa, J. Carlos Menéndez

**Affiliations:** a Unidad de Química Orgánica y Farmacéutica , Departamento de Química en Ciencias Farmacéuticas , Facultad de Farmacia , Universidad Complutense , 28040 Madrid , Spain . Email: josecm@farm.ucm.es

## Abstract

Historically, the use of mechanochemical methods in synthesis has been almost negligible, but their perception by the synthetic community has changed in recent years and they are on their way to becoming mainstream. However, the hybridization of mechanochemical synthesis with methodologies designed to increase synthetic efficiency by allowing the generation of several bonds in a single operation has taken off only recently, but it already constitutes a very promising approach to sustainable chemistry. In this context, we provide in this Perspective a critical summary and discussion of the main known synthetic methods based on mechanochemical multicomponent reactions.

## Abstract 2

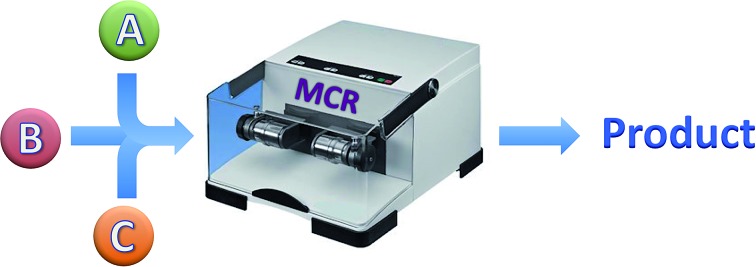
Multicomponent reactions promoted by mechanical energy are critically reviewed.

## 1. Introduction

Mechanochemistry is characterized by the application of mechanical energy (*e.g.* by compression, shear, or friction) to achieve chemical transformations. It has a variety of applications in areas as diverse as nanoscience or engineering of minerals, but these aspects will not be treated here. Furthermore, it allows performing chemical reactions, serving as a complement to traditional strategies based on thermal or irradiative activation. Thus, the IUPAC Compendium of Chemical Technology (“gold book”) defines mechanochemical reactions as those that are induced by the direct absorption of mechanical energy, which may come from grinding or milling processes.

Mechanochemical activation is of particular significance in the context of green chemistry because it allows the use of solvent-free conditions. It is important to notice that volatile organic solvents constitute the main type of residues from synthetic chemistry, both at laboratory and industrial scales. Working under solvent-free conditions, solvation phenomena are not relevant and this often leads to accelerated reactions. Another factor allowing improved reaction rates is the fact that the reactions are performed at very high reagent concentrations owing to the absence of solvents. These very peculiar conditions may lead to alterations in product selectivity.^1^ Furthermore, there is much experience in the application of ball milling at industrial scale to achieve reduction in particle size during the manufacturing of drugs, paints and other products and therefore the scale-up of mechanochemical synthetic protocols is feasible.^2^

**Fig. 1:**
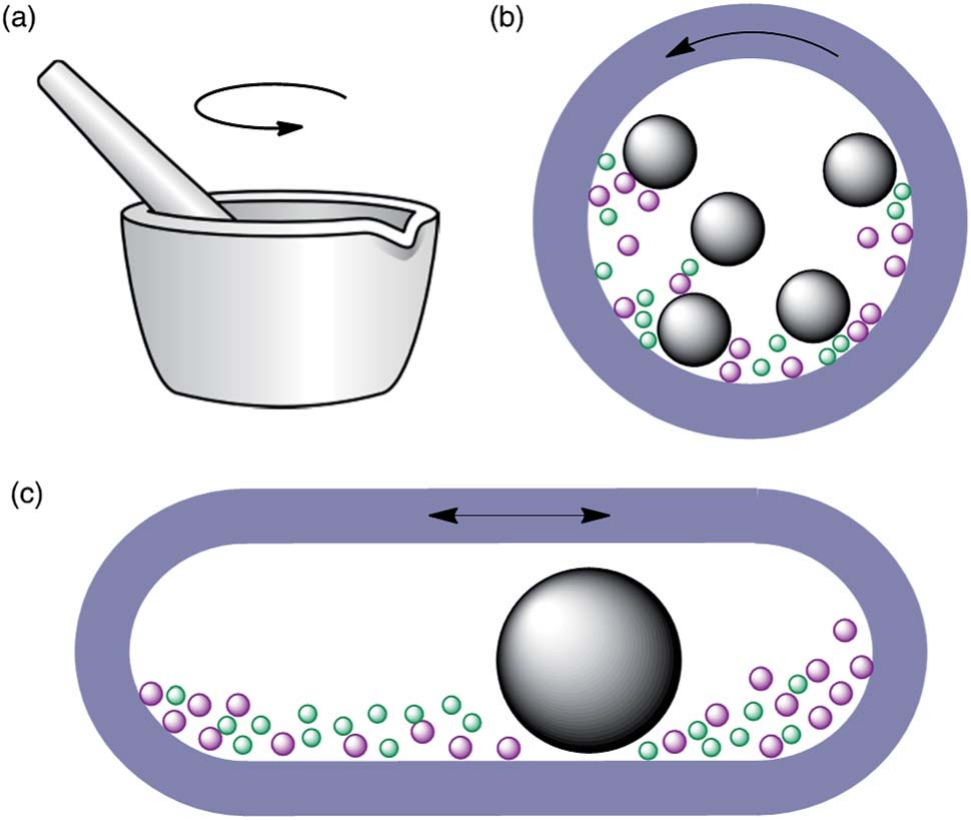
Schematic representations of the three main modes of mechanical activation of chemical reactions: (a) grinding with mortar and pestle (“grindstone” activation); (b) planetary ball milling; (c) high-speed vibration milling in a mixer mill.

Historically,^3^ the first mechanochemical reactions were achieved by grinding reactants together with a mortar and pestle, an approach that is sometimes referred to as “grindstone chemistry”. While this technique does not require specialized equipment and is therefore easy to perform in any laboratory, it has the limitations of not being practical unless reaction times are short and not being always easy to reproduce, as it is dependant on the physical strength of the operator. More recently, automated ball mills have been introduced for laboratory-scale synthesis. These instruments allow the control of energy input by adjusting the milling frequency, and therefore have better reproducibility. Furthermore, they are safer as the reactions are performed in closed vessels and the operator is not exposed to the reactants, catalysts or products. Two main types of instruments are available, namely planetary ball mills and mixer (shaker) mills (Fig. 1). In the former, the balls and reactants experience two types of movements, namely friction with the inside walls of the jar as a result of the centrifugal force and impact when they lift off and collide with the opposite wall. In mixer mills, the jar is placed horizontally and swings back and forth, a movement that causes the balls and reactants to collide with the opposite wall of the jar and is usually described as high-speed vibration milling (HSVM) or high-speed ball milling (HSBM). The main factors that influence these reactions, besides the obvious ones such as the reaction time and milling frequency, are the type of ball mill, the material of milling balls and jars and the number of balls.

In order to increase friction, specially for liquid starting materials, it may be necessary to include a solid that allows the transfer of mechanical energy.^4^ Common milling auxiliaries are NaCl, Al_2_O_3_ and SiO_2_, and they may be inert under the reaction conditions or assist the reaction; for instance, SiO_2_ provides an acidic environment and it also retains water, thereby displacing condensation equilibria in some cases.^5^

Striking accelerations of mechanochemical reactions have been observed upon addition of small amounts of liquids to the solid reacting mixtures, a phenomenon that has been described as solvent-drop grinding (SDG) or liquid-assisted grinding (LAG). The expression “ionic liquid-assisted grinding” (ILAG) has also been used, when the acceleration comes from the addition of an ionic liquid. Polymer-assisted grinding (POLAG) is another recently introduced variation of mechanochemistry.^6^

Ball milling is a batch processing technique and, as such, it has some inherent limitations when used at high reaction scales such as long idle times and problems with product isolation when the material to be obtained is not a free flowing powder. For this reason, it would be important, particularly in industrial settings, to have access to mechanochemical methods that can be adapted to working in flow conditions. The term extrusion describes a variety of continuous processing techniques that achieve the intense mixing of materials by forcing them through constrained spaces, through compression forces and shear. These methods constitute an alternative approach to mechanochemical synthesis that can be performed as a flow process and thus be complementary to ball milling. Extrusion has traditionally been used in the polymer, pharmaceutical and food industries, but more recently it has also found application in the field of mechanochemical synthesis, in particular for the preparation of metal–organic frameworks and co-crystals,^7^ but also in some examples of more conventional organic synthetic transformations.^8^

**Fig. 2:**
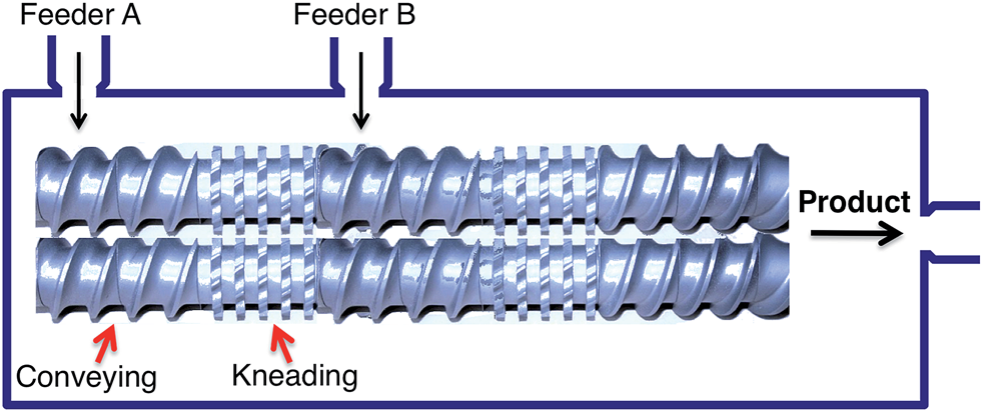
A schematic twin screw extrusion setup.

The most common way to achieve the movement of materials into a confined space is to force them along a screw, and there are two main types of processes based on this principle, namely single (SSE) and twin screw extrusion (TSE), the latter being more commonly employed. In TSE, solid reactants are conveyed through a barrel by the movement of two intermeshing, counter-rotating screws. The screws create a series of alternating conveying and kneading segments in such way that the overall system configuration can be adjusted for each particular transformation (Fig. 2).

### 1.1. Multicomponent reactions and their importance in contemporary synthetic chemistry

There is a growing interest in synthetic methods that allow the fast and experimentally simple preparation of compound libraries for research programs aimed at the discovery of new chemical entities for the pharmaceutical and agrochemical industries.^9^ In this context, reactions that generate several bonds in a single operation (multiple bond-forming transformations, MBFTs)^10^ are gaining importance because of their high synthetic efficiency and the fact that they reduce the number of isolation and purification steps and hence the generation of waste from organic solvents and discarded chromatographic stationary phases.^11^ Among the various types of MBFTs, multicomponent reactions (MCRs) have received much attention in recent years. They are defined as processes in which three or more starting materials are combined, either by mixing them simultaneously or by their sequential addition to the reaction medium, to form a product that contains significant structural fragments of all reactants.^12^

### 1.2. Mechanochemical multicomponent reactions

Mechanochemical synthesis has experienced an explosive growth in recent years and has been the subject of a considerable number of reviews,^13^ a monograph^14^ and several journal themed issues.^15^ However, its combination with other strategies that achieve improved synthetic efficiency and a diminished generation of solvent waste has been relatively neglected and has not been reviewed previously. Such type of combination, which leads to synergy between the advantages of both approaches and can be expected to become soon one of the frontiers in green synthetic methodology, is the subject of the present Perspective article. As will be discussed in Section 8, additional advantages such as improved yields, diminished reaction times and others are often achieved by application of mechanochemical activation to MCRs.

## 2. Mechanochemical multicomponent reactions for the synthesis of acyclic compounds

### 2.1. Isocyanide-based multicomponent reactions

**Scheme 1:**
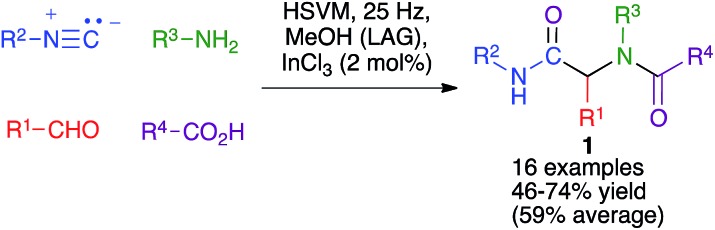
The mechanochemical Ugi reaction.

The Ugi four-component reaction from isonitriles, primary amines, aldehydes and carboxylic acids is a very important tool in the construction of diversity-oriented compound libraries. A mechanochemical version of this transformation has been developed by Juaristi, and is based on the use of high-speed vibration milling with a single agate ball (6 mm diameter) in an agate jar and methanol as a liquid-assisted grinding agent to furnish compounds **1**. The scope of the method was tested mainly for aromatic aldehydes (Scheme 1).^16^

**Scheme 2:**
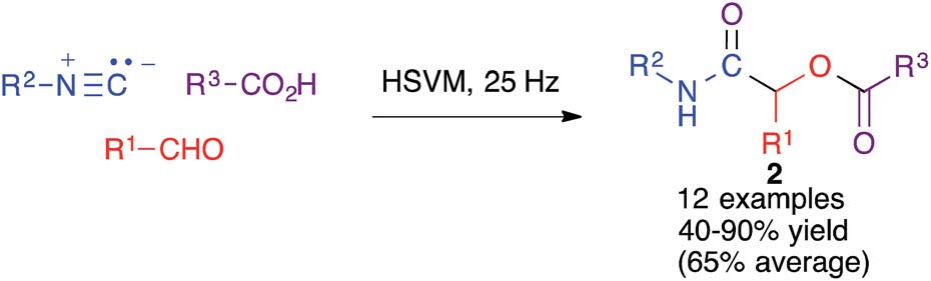
The mechanochemical Paserini reaction.

The same group studied the mechanochemical Passerini reaction, another important isocyanide-based MCR leading to compounds **2** (Scheme 2). These reactions were performed in a stainless steel jar, with two 6 mm balls of the same material.

### 2.2. Other multicomponent transformations initiated by the formation of an imine

**Scheme 3:**
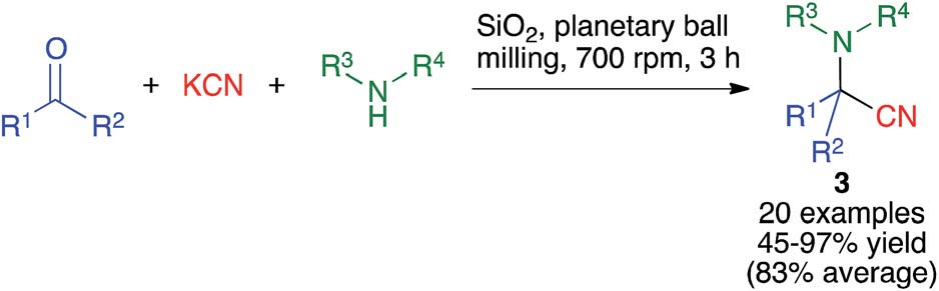
Strecker reactions performed under planetary ball milling.

The Strecker reaction between aldehydes or ketones, amines and cyanide to furnish α-aminonitriles was the first reported multicomponent reaction and hence a milestone in the development of organic synthesis.^17^ Hernández and Bolm have recently disclosed their work on the mechanochemical Strecker reaction (Scheme 3), employing a planetary ball mill operating at 700 rpm and containing 20 agate milling balls (5 mm diameter). They found that the use of silica gel as a milling auxiliary was highly beneficial, probably due to its acidity coupled with its ability to displace the equilibrium leading to the formation of the intermediate imine by absorbing the generated water^5^ The reaction showed a very good substrate scope, being able to accomodate the use of either aldehydes or ketones (with the exception of benzophenone) as the carbonyl component and ammonia, primary aliphatic, primary aromatic and secondary aliphatic amines and also a sulfonamide, albeit in lower yield. The same group later demonstrated the use of kraft lignin, an example of readily available lignocellulosic biomass, as a suitable additive to promote the mechanochemical Strecker reaction.^18^

**Scheme 4:**
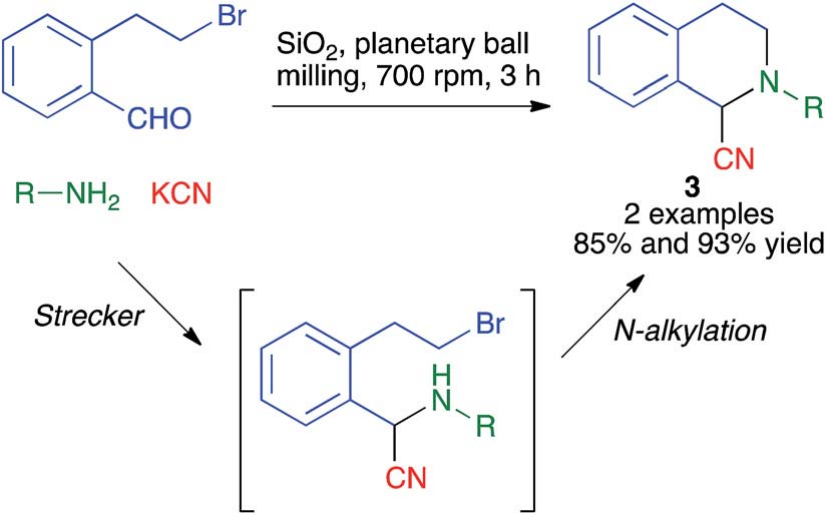
A mechanochemical one-pot Strecker/intramolecular *N*-alkylation domino sequence leading to tetrahydroisoquinolines.

Interestingly, the mechanochemical conditions could also be adapted to the three-component synthesis of tetrahydroisoquinolines **3***via* a one-pot Strecker reaction/intramolecular *N*-alkylation domino sequence (Scheme 4).

**Scheme 5:**
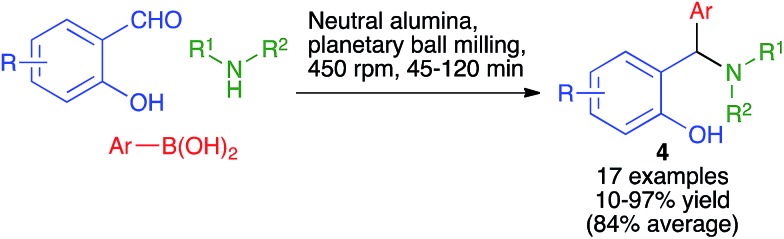
Petasis reactions performed under planetary ball milling.

Hosseinzadeh and coworkers have developed a solvent-free mechanochemical protocol for the Petasis reaction, a variation of the Mannich reaction where a boronic acid acts as the nucleophile. Thus, the reaction between salicylaldehyde derivatives, primary or secondary amines and arylboronic acids in a planetary ball mill containing stainless steel balls (2 balls, 1 cm diameter) and operating at 450 rpm afforded the *ortho*-substituted phenol derivatives **4** (Scheme 5). The method normally gave excellent results, but the method failed or gave poor yields in some examples involving the use of primary aromatic amines unless they carried electron-withdrawing groups, and also in the case of diphenylamine.^19^ The Petasis reaction has also been performed under twin screw exclusion.^8^

**Scheme 6:**
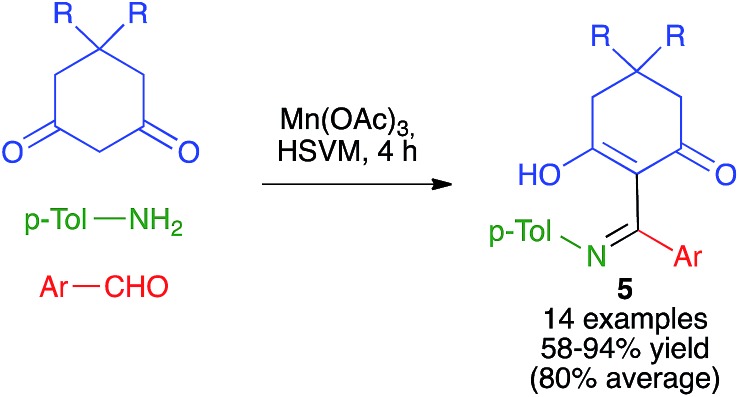
A radical Mannich-like oxidative process under high-speed vibration milling.

As summarized in Scheme 6, Zhang and coworkers have developed a Mannich-like oxidative addition of carbon radicals affording compounds **5** from 1,3-cyclohexanedione derivatives in the presence of manganese(iii) acetate and *in situ*-generated imines under high-speed vibration milling conditions, using a single stainless steel ball (7 mm diameter) in a jar of the same material.^20^

The three-component reaction between aromatic aldehydes, terminal alkynes and anilines, performed in the presence of 10 mol% of copper(ii) triflate and Ph-Pybox (**6**) as a chiral ligand, afforded chiral propargylamines **7** in excellent yields and enantiomeric excesses *via* an enantioselective A3 coupling.

**Scheme 7:**
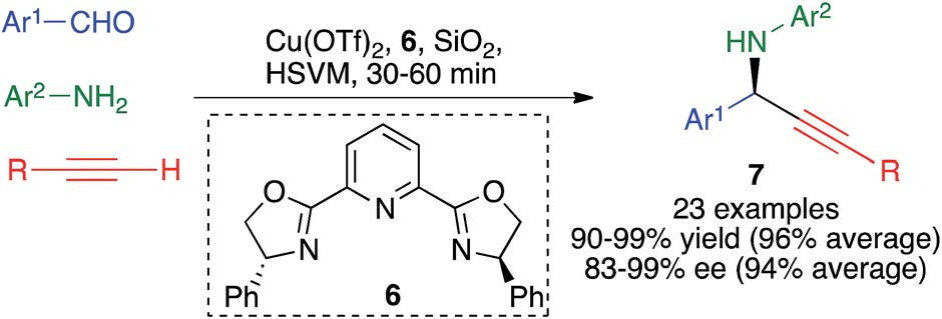
An enantioselective mechanochemical A3 coupling.

The optimized experimental conditions developed for this transformation involved the use of stainless steel balls (2 balls, 1.5 cm diameter) in a jar of the same material under high-speed vibration ball-milling at 30 Hz, in the absence of solvent and using silica gel as a grinding auxiliary (Scheme 7).^21^ It was also verified that a single dichloromethane extraction of the crude reaction product followed by evaporation allowed the recovery of the catalyst, which was fully functional for up to four subsequent runs of the reaction.

### 2.3. Synthesis of ureas and thioureas

**Scheme 8:**
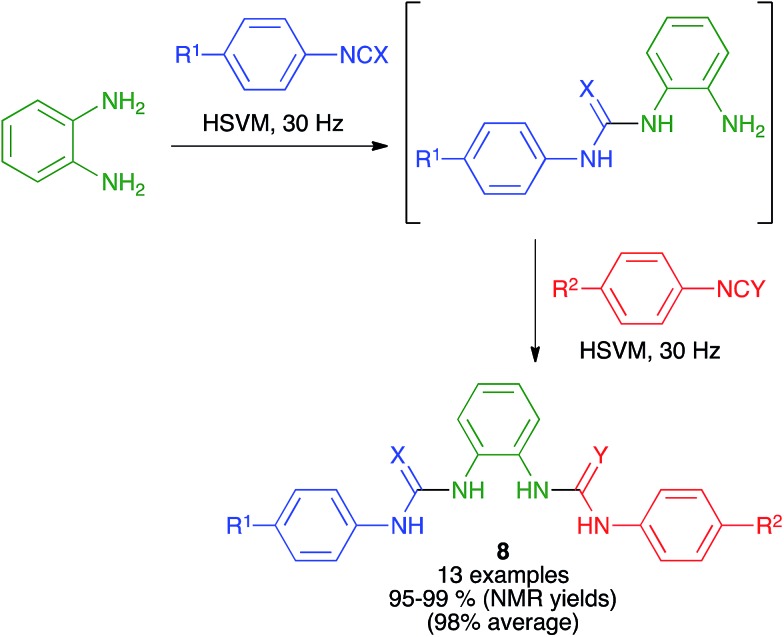
Synthesis of ureas (X = O) and thioureas (X = S) *via* the desymmetrization of *o*-phenylenediamine under mechanochemical conditions.

Urea and thiourea derivatives are important in drug discovery, and also as chiral organocatalysts. As shown in Scheme 8, Eckert-Maksić and Friščić demonstrated the generation of non-symmetrical ureas and thioureas **8** from *o*-phenylenediamine by sequential addition of two different isocyanates or isothiocyanates under high-speed vibration milling conditions, employing a single 12 mm diameter stainless steel ball at a frequency of 30 Hz. The intermediate products from the initial addition could be isolated if desired, and the method could also be applied to *p*-phenylenediamine as the starting material.^22^

**Scheme 9:**
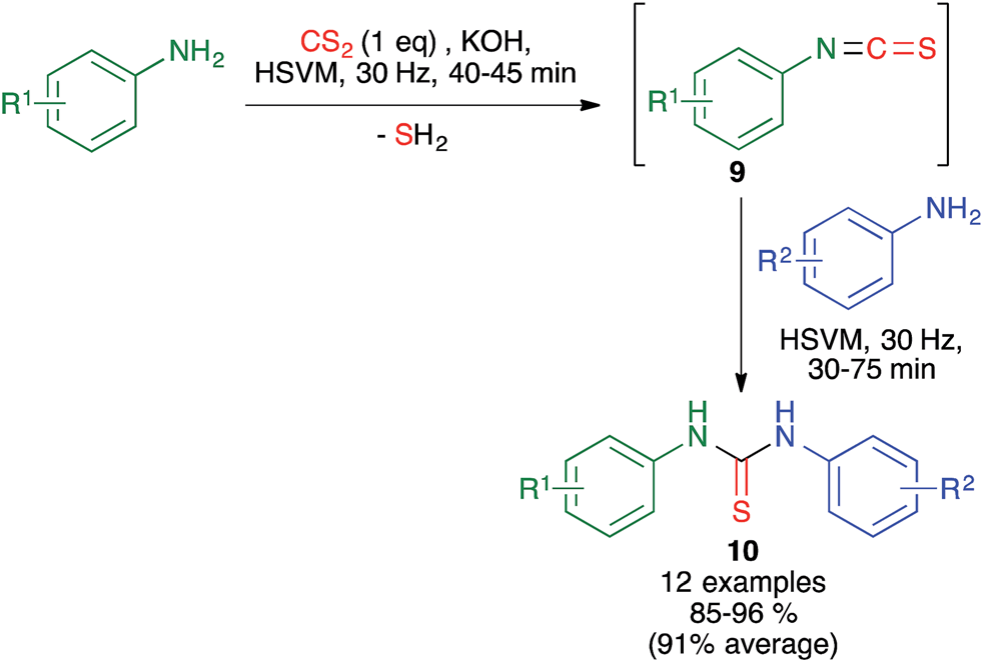
Mechanochemical generation of isothiocyanates and related sequential three-component synthesis of non-symmetrical diarylthioureas.

In related work, the Zhang group proved that the starting isothiocyanates **9** could also be generated by ball milling at 30 Hz, starting from anilines and CS_2_ in the presence of KOH. Addition to this mixture of a second aniline and further ball milling allowed the preparation of unsymmetrical thioureas **10** (Scheme 9). The isothiocyanates could be isolated by interrupting the process after the first step.^23^

### 2.4. Synthesis of dithiocarbamates

**Scheme 10:**
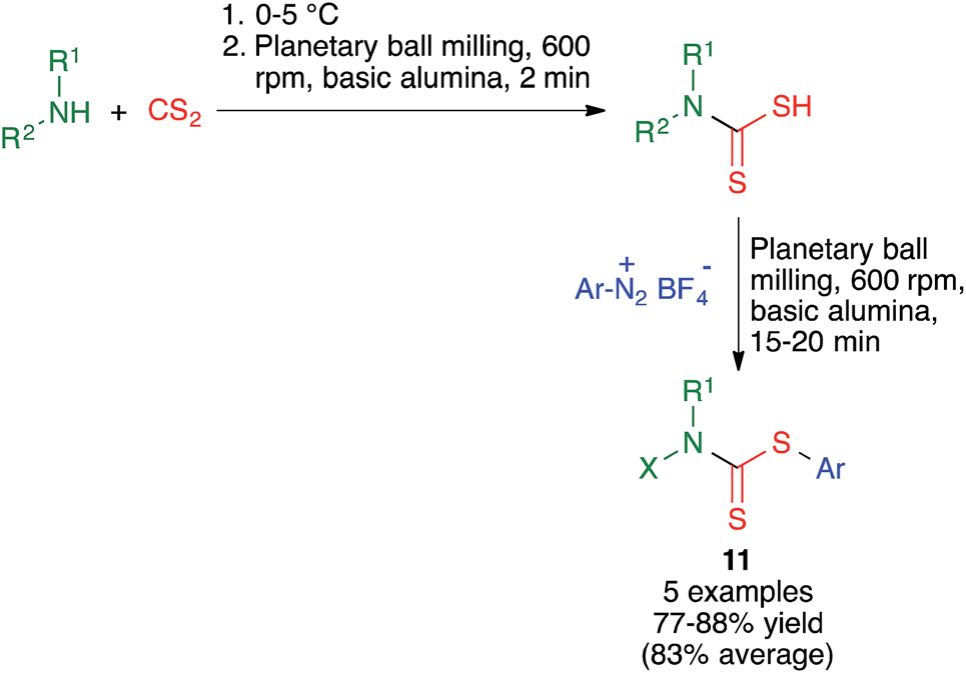
Multicomponent synthesis of dithiocarbamate under planetary ball milling conditions.

Ranu and coworkers have disclosed a solvent-free synthesis of *S*-aryl dithiocarbamates **11** by the three-component reaction of aryldiazonium fluoroborate derivatives, carbon disulfide and amines (Scheme 10). The reaction was performed by first mixing the starting amine and carbon disulfide at at 0–5 °C followed by brief milling for 2 min in a planetary ball mill operating at 600 rpm and containing six balls (10 mm in diameter) in the presence of basic alumina. The suitable diazonium salt was then added and ball milling was continued for an additional period of 15–20 min.^24^

## 3. Mechanochemical multicomponent reactions for the synthesis of benzene rings

**Scheme 11:**
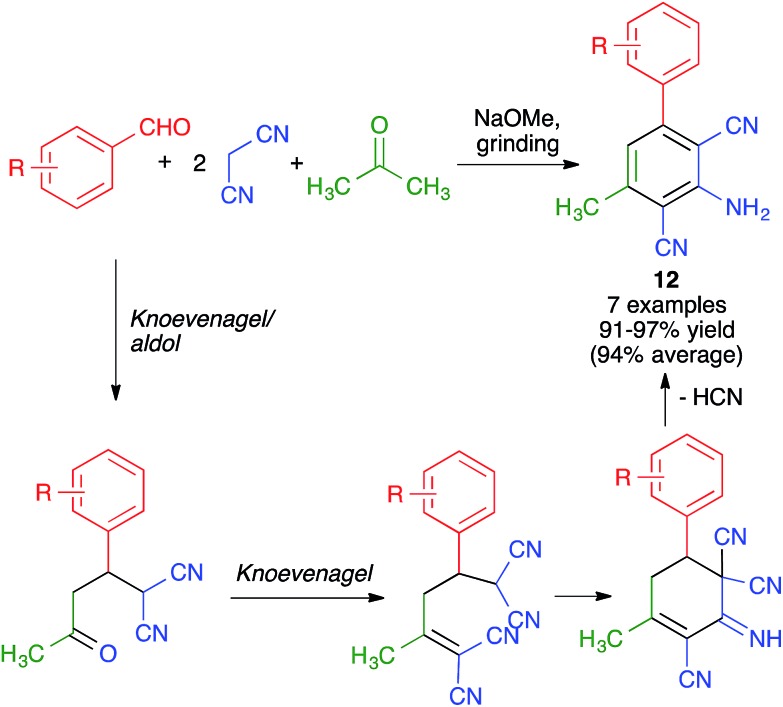
Synthesis of biphenyls by generation of one of its benzene rings using a mechanochemical MCR.

Pasha and Datta synthesized highly functionalized biphenyl systems **12***via* the generation of one of their benzene rings using a manual grinding-assisted multicomponent reaction from aromatic aldehydes, malononitrile and acetone in the presence of a catalytic amount of sodium methoxide.^25^ The method gave excellent yields but only allowed structural variations in one of the phenyl rings, where either electron-withdrawing or electron-releasing substituents were tolerated. This complex transformation was proposed to take place by a domino sequence comprising Knoevenagel, aldol, Knoevenagel, intramolecular condensation, HCN elimination and imine–enamine tautomerism steps (Scheme 11).

## 4. Mechanochemical multicomponent reactions for the synthesis of heterocycles

We have organized this part of the Perspective according to the size of the ring generated in the multicomponent reaction, with no effort to differentiate systems having a single ring from fused or spiro compounds.

### 4.1. Five-membered heterocycles

#### Furan derivatives

**Scheme 12:**
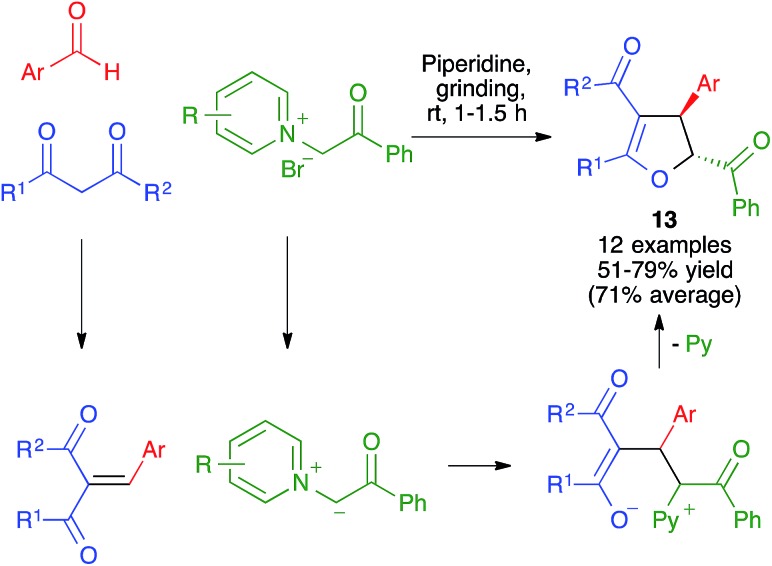
Diastereoselective three-component synthesis of *trans*-2,3-disubstituted 2,3-dihydrofurans by manual grinding.

Chuang and Chen have studied the diastereoselective synthesis of *trans*-2,3-disubstituted 2,3-dihydrofurans **13** from enolizable 1,3-dicarbonyl compounds, aromatic aldehydes and *N*-phenacylpyridinium bromides, which acted as ylide precursors. They initially developed the reaction under conventional solution conditions, but eventually discovered that it could also be performed by manually grinding the starting materials in a mortar at room temperature in the presence of piperidine, acting as a base (Scheme 12).^26^

#### Thiophene derivatives

**Scheme 13:**
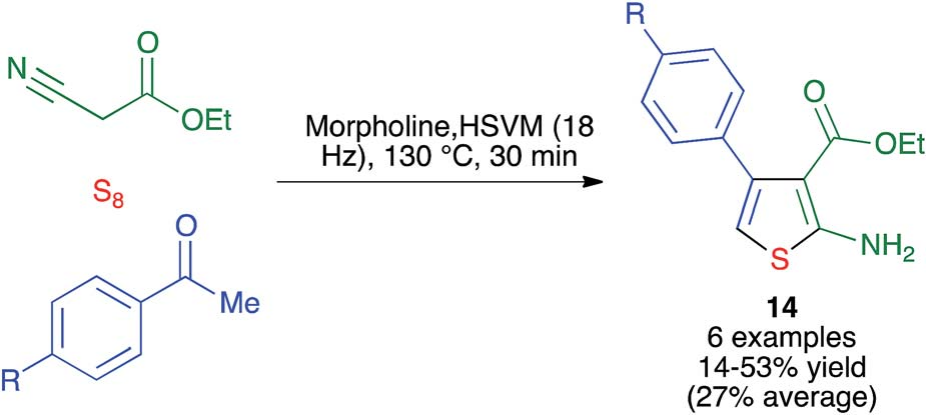
The mechanochemical Gewald thiophene synthesis.

The Gewald reaction of ketones with α-methylenecarbonyl compounds, activated nitriles and elemental sulfur is one of the most convenient methods for preparing densely substituted 2-aminothiophenes, which are of interest in medicinal chemistry, although it is slow for some carbonyl substrates such as aryl ketones. Mack and coworkers discovered that the Gewald reaction could be performed under ball milling conditions with a stainless steel ball (1/8′′) at 18 Hz in the presence of a catalytic amount of base to furnish thiophene derivatives **14**. Using a mixer mill modified to allow fitting a heat gun, they carried out their reactions at 130 °C, finding improved results over the purely thermal conditions in the reactions of acetophenone derivatives, although the yields were still moderate (Scheme 13).^27^

#### Pyrroles and their fused and spiro derivatives

**Scheme 14:**
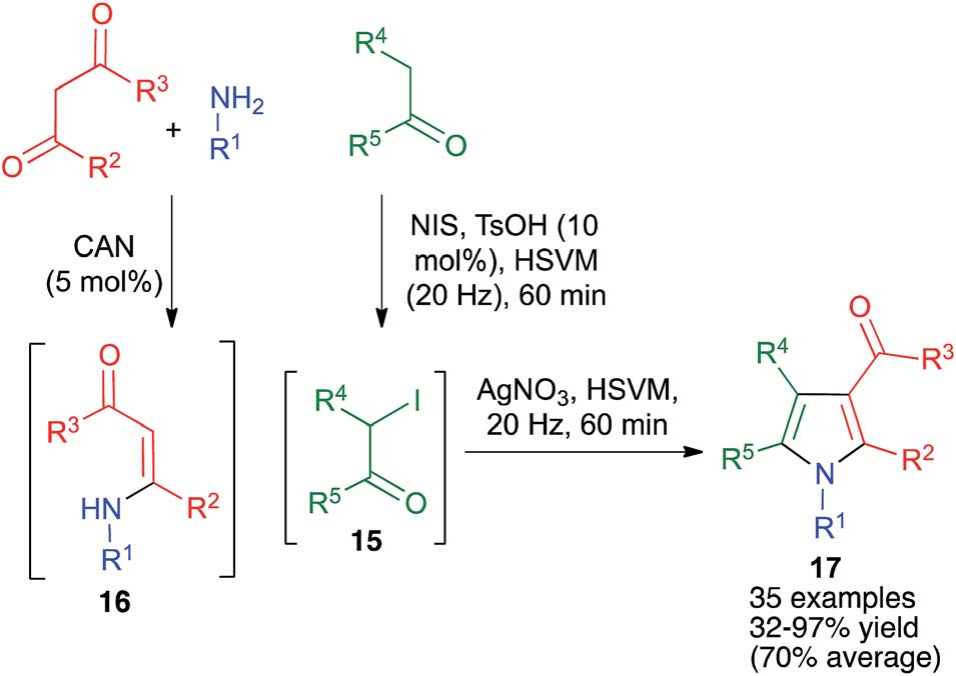
A generalization of the Hantzsch pyrrole synthesis under high-speed vibration milling conditions.

The Hantzsch pyrrole synthesis from α-haloketones and β-enaminones, which may be generated *in situ* in a three-component fashion, is one of the most traditional methods for pyrrole synthesis. However, in spite of its named reaction status, the conventional Hantzsch pyrrole synthesis has many limitations in scope and is far from being a general process. Some years ago, we discovered that by performing the reactions under high-speed vibration milling conditions in a mixer mill operating at 20 Hz and using a single zirconium oxide ball 20 mm in diameter, it was possible to design a sequential telescoped process combining the α-iodination of ketones with *N*-iodosuccinimide (NIS) in the presence of acid to give **15** and the three-component Hantzsch pyrrole synthesis *via* the formation of the intermediate enaminone **16**. This transformation was performed using Ce(iv) ammonium nitrate (CAN) as catalyst and an equimolecular amount of silver nitrate, which was necessary to prevent reductive dehalogenation of **15** by the iodide anion liberated during the alkylation step (Scheme 14). This method was compared with a similar protocol performed in solution, starting from isolated α-iodoketones, and it was found that the mechanochemical method afforded significantly higher yields of pyrroles **17** in spite of comprising an additional step, being far more general than previous versions of the Hantzsch reaction.^28^,^29^

**Scheme 15:**
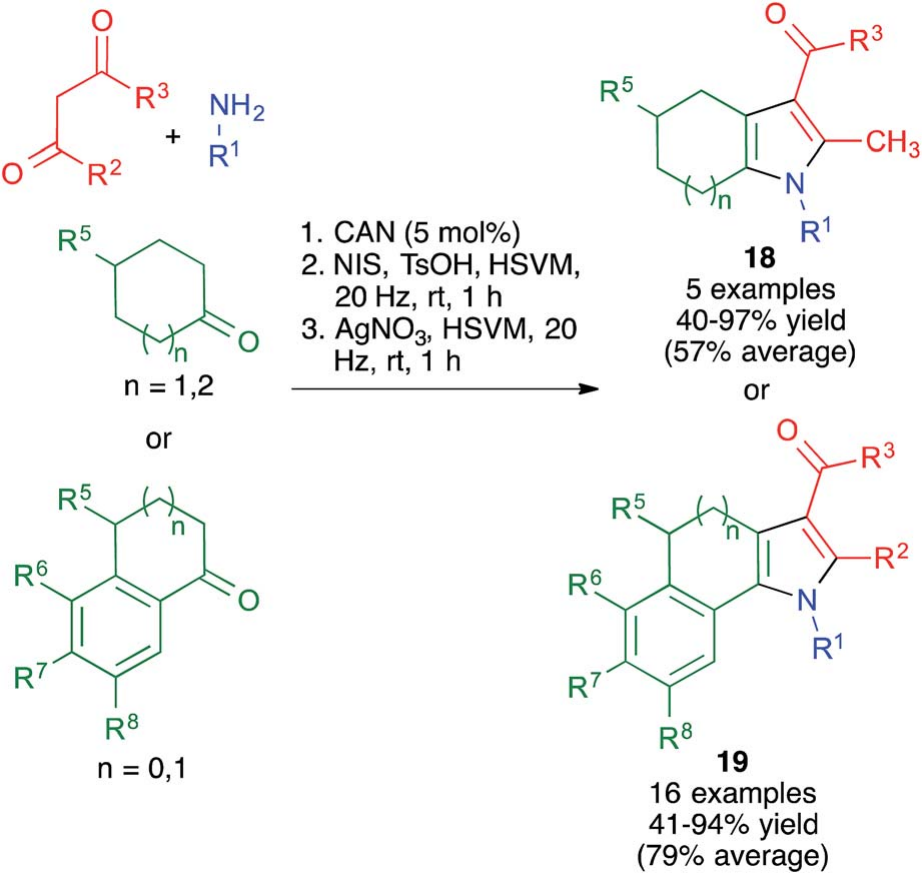
Mechanochemical synthesis of fused pyrrole derivatives.

As shown in Scheme 15, the broad scope of this method was shown by its application to the synthesis of fused pyrrole derivatives derived from the indole, homoindole, benzo[*g*]indole and indeno[1,2-*b*]pyrrole frameworks (compounds **18** and **19**), which had not been previously achieved using Hantzsch chemistry. Again, the mechanochemical method proved to have considerable advantages in terms of yield over a similar solution-phase protocol.^29^

The mildness of the conditions leading to the pyrrole derivatives encouraged us to attempt the use of starting materials bearing additional functional groups, which were hoped not to interfere with the Hantzsch-type process but to be later amenable to a cyclization reaction, thus affording structurally complex frameworks in only two steps. The method for the generation of diversity-oriented libraries consisting of the combination of a multicomponent reaction with a subsequent complexity-generating event was proposed by Schreiber, who called it the build-couple-pair strategy.^30^

**Scheme 16:**
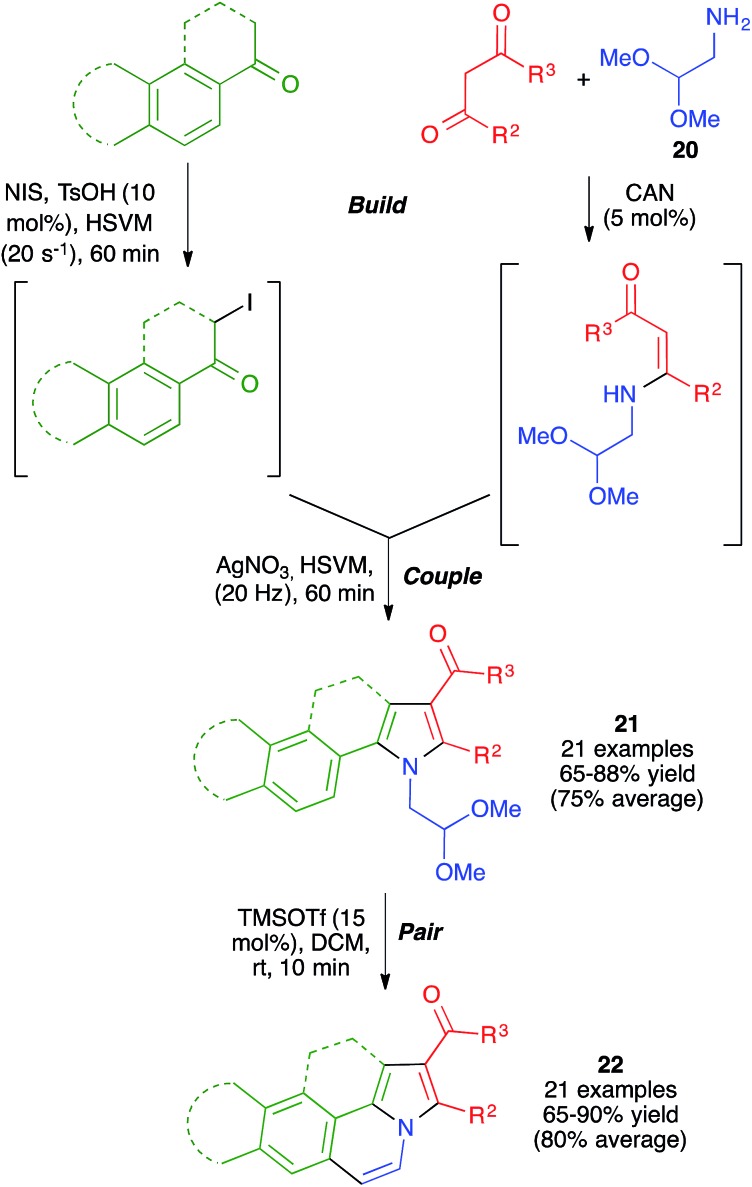
Application of the mechanochemical Hantzsch pyrrole synthesis to the generation of structurally diverse polyheterocyclic systems using the build-couple-pair strategy.

With these ideas in mind, we examined the use of 2-aminoacetaldehyde dimethylacetal **20** as the starting material for our mechanochemical Hantzsch pyrrole synthesis (Scheme 16). Interestingly, the mildness of the reaction conditions and the absence of solvent prevented the CAN-promoted hydrolysis of the acetal functional group and the pyrrole and fused pyrrole derivatives **21** were obtained uneventfully. For the cyclization step, we discovered that the use of trimethylsilyl triflate in catalytic amounts allowed performing Pommeranz–Fritzsch-type cyclizations affording polycyclic compounds **22** in high yields at room temperature.^31^

**Scheme 17:**
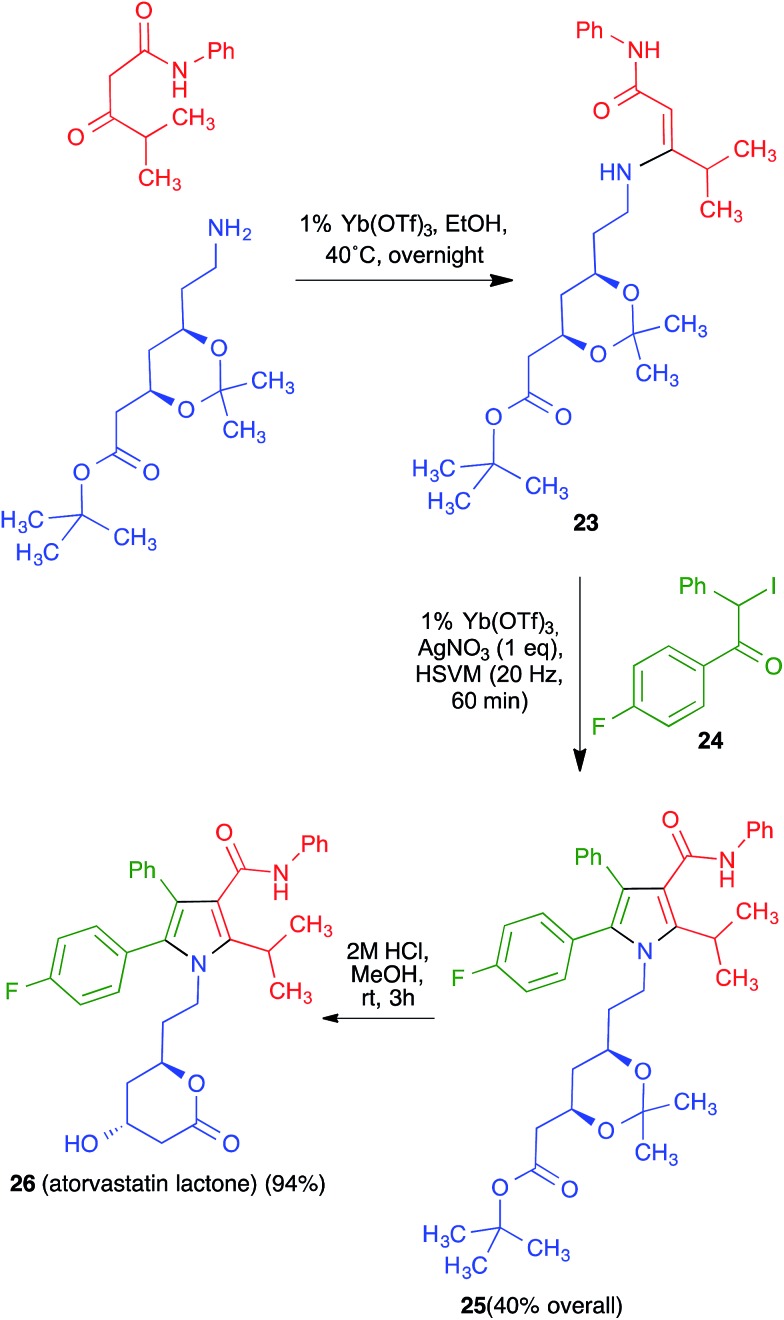
Synthesis of atorvastatin lactone based on the mechanochemical Hantzsch pyrrole synthesis.

Having proved its usefulness in a diversity-oriented context, we made the decision to apply the mechanochemical Hantzsch pyrrole synthesis to a target-oriented problem, prompted by the fact that the synthesis of complex pyrrole-derived synthetic targets, specially in an atom- and step-economic fashion, is still challenging. Because the antihyperlipidemic agent atorvastatin is arguably the most important pyrrole-based bioactive molecule, having been the top-selling drug for more than a decade, it was chosen as the target for this project. Previous syntheses of the atorvastatin pyrrole core normally have as key steps the classical Paal–Knorr pyrrole synthesis or 1,3-dipolar cycloadditions,^32^ but our method furnished an opportunity to develop a very concise, convergent route to this system (Scheme 17). Although the enaminone-forming step failed under the previously developed conditions, we discovered that ytterbium triflate was a suitable catalyst, although the reaction was slow and had to be performed at 40 °C overnight. A mixture of the crude enaminone **23** and compound **24** was submitted to high-speed vibration milling at 20 Hz for 1 h in the presence of silver nitrate, leading to **25** in 40% overall yield. A final hydrolytic deprotection with concomitant acid-promoted lactonization afforded a 94% yield of the so-called atorvastatin lactone **26**,^33^ which can be easily transformed into the final drug molecule by hydrolysis and salt formation using literature methods. It is worth emphasizing that, by taking advantage of the very concise nature of multicomponent reactions, the Hantzsch-based route led to the shortest synthesis of the atorvastatin lactone published to date.

**Scheme 18:**
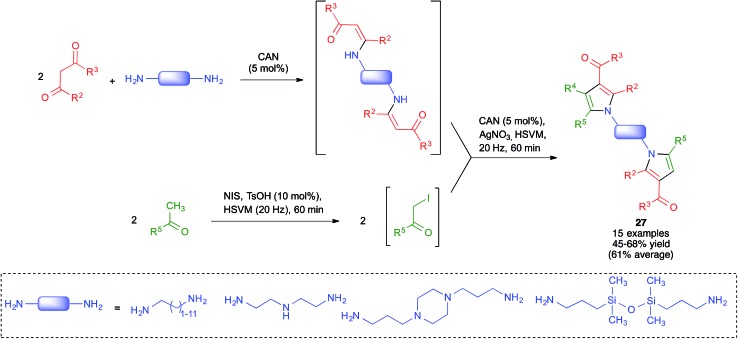
Mechanochemical pseudo-five component reactions starting from diamines and leading to symmetrical systems containing two pyrrole units.

**Scheme 19:**
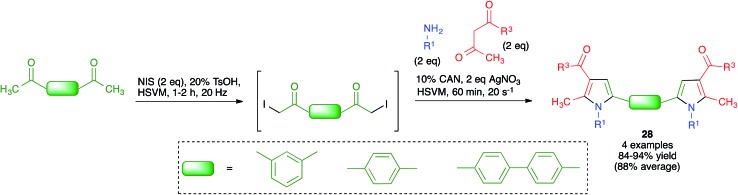
Mechanochemical pseudo-five component reactions starting from diacetylarenes and leading to symmetrical systems containing two pyrrole units.

Because of the importance of symmetrical molecules formed by two or more pharmacophoric units joined by a spacer for drug discovery programs, the ability to build two identical heterocyclic systems at both ends of a spacer chain in a single operation is important for medicinal chemists. For this reason, we studied the pseudo-five component reactions between β-dicarbonyl compounds (2 eq.), diamines and α-iodoketones (prepared *in situ* from aryl ketones, 2 eq.), under our previously established mechanochemical conditions. We found that the target compounds **27** were readily accessible, although in diminished yields compared to their simpler analogues **17**, probably owing to the fact that the formation of **27** involves seven individual steps (Scheme 18).^34^ Similarly, symmetrical aromatic compounds containing two acetyl groups were also employed as starting materials for pseudo-five component reactions leading to systems containing two pyrrole units joined by a spacer (compounds **28**) (Scheme 19).^29^

**Scheme 20:**
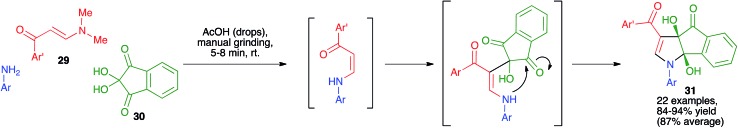
Three component mechanochemical synthesis of dihydroindeno[1,2-*b*]pyrrole derivatives.

As shown in Scheme 20, other fused pyrroles that have been prepared by mechanochemical techniques include the dihydroindeno[1,2-*b*]pyrrole derivatives **31**. These structurally interesting compounds were synthesized as single diastereomers by Perumal and coworkers by simply grinding for short times equimolecular amounts of (*E*)-3-(dimethylamino)-1-arylprop-2-en-1-ones **29** with anilines and ninhydrin **30** in the presence of a small amount of acetic acid, and were isolated in pure state without the need for chromatographic purification.^35^

**Scheme 21:**
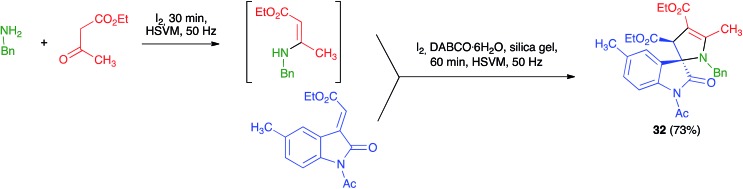
Mechanochemical synthesis of 3,2′-pyrroline-spirooxindoles from *in situ*-generated β-enaminoesters.

Xu *et al.* described a mechanochemical protocol under high-speed vibration milling for the diastereoselective preparation of 3,2′-pyrroline-spirooxindoles **32** under solvent-free conditions. In general, their method was not multicomponent because they started from isolated β-enamino esters and alkylidene oxindoles that were combined in the presence of I_2_, DABCO·H_2_O and using silica gel as milling auxiliary. Nevertheless, they also examined one example of a three-component variation of their reaction, which gave only slightly lower yield than their standard protocol, as shown in Scheme 21. The reaction was performed in a 3.5 mL stainless steel jar containing 8 stainless steel balls of 5 mm in diameter and working at a frequency of 50 Hz, at room temperature during 60 minutes.^36^

#### Pyrazoles

**Scheme 22:**
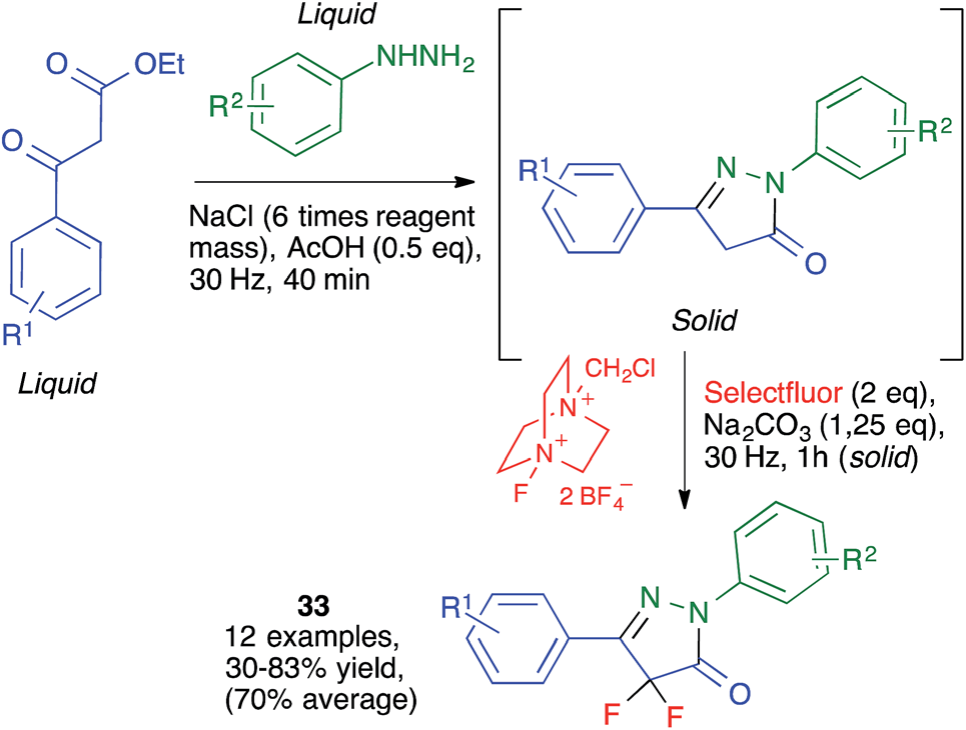
Synthesis of fluorinated pyrazolones by high-speed vibration milling.

Browne and co-workers have reported a solvent-free, one-pot mechanochemical synthesis of a library of pharmacologically relevant difluorinated pyrazolones **33** by HSVM in a 10 mL stainless steel milling jar containing a single stainless steel ball (10 mm in diameter) and operating at 30 Hz. Their work illustrates the use of sodium chloride as a grinding auxiliary, which was necessary in the first step because of the liquid nature of both reagents (Scheme 22).^37^

#### Oxazoles, thiazoles and their benzo derivatives

**Scheme 23:**
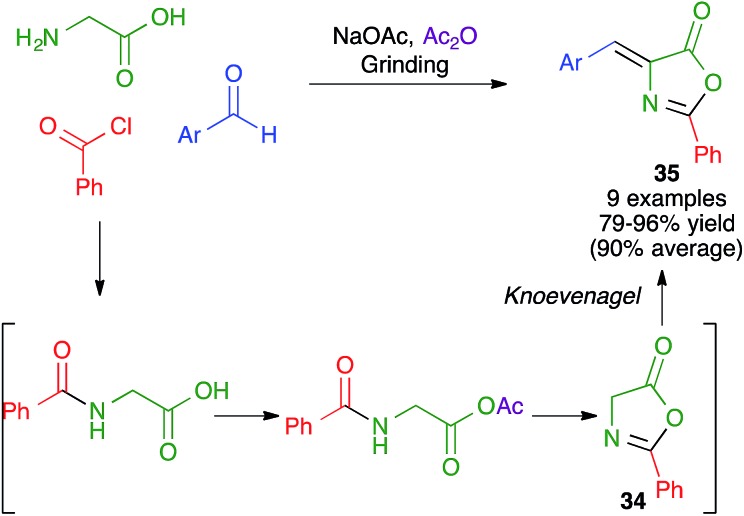
Mechanochemical synthesis of azlactones and their *in situ* Knoevenagel condensations.

5(4*H*)-Oxazolones, known as azlactones, have a number of applications in materials science and medicinal chemistry and also are important intermediates in the synthesis of amino acids, peptides and several heterocyclic systems. Fahmy *et al.* have described a multicomponent one-pot synthesis of 4-arylidene-2-phenyl-5(4*H*)-oxazolones **35** that combines the preparation azlactones **34** with their Knoevenagel condensation with aldehydes (Scheme 23). This method involves the solvent-free grinding in a mortar at room temperature of a mixture of glycine, benzoyl chloride, an aromatic aldehyde and fused sodium acetate in the presence of acetic anhydride. The mechanism is initiated by the benzoylation of glycine to give hippuric acid, followed by activation of the latter as a mixed anhydride with subsequent cyclization to azlactone **34**, and a final Knoevenagel condensation with the aromatic aldehyde.^38^

**Scheme 24:**
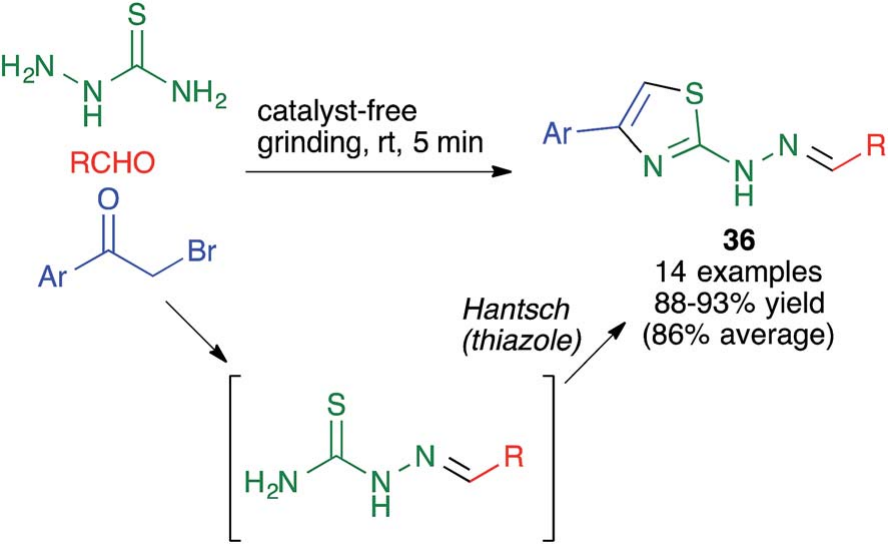
Mechanochemical combination of a thiosemicarbazone formation with a Hantzsch thiazole synthesis.

Another mechanochemical combination of a heterocyclic synthesis with a condensation reaction was employed by Whu and coworkers to achieve an efficient, catalyst- and solvent-free synthesis of pharmacologically relevant 4-aryl-2-thiazolylhydrazones **36** by a one-pot reaction performed by manually grinding aldehydes, α-bromoketones and thiosemicarbazide at room temperature. This transformation was assumed to proceed by the initial formation of a thiosemicarbazone, which would be followed by a Hantzsch thiazole synthesis (Scheme 24); the alternative Hantzsch/condensation pathway was discarded due to the fact that thiosemicarbazide and α-bromoketones failed to react under the reaction conditions.^39^

**Scheme 25:**
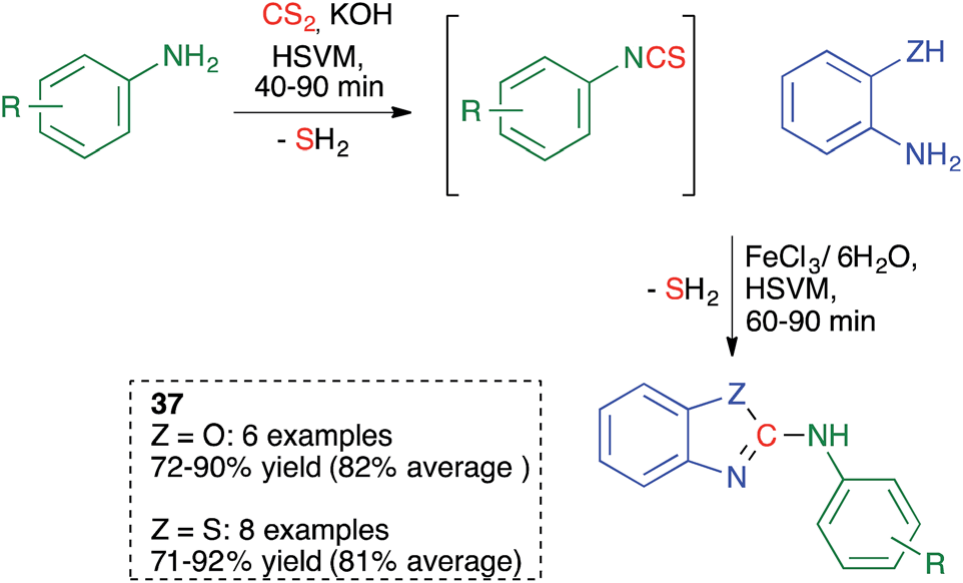
Mechanochemical synthesis of benzoxazoles and benzothiazoles.

As summarized in Scheme 25, Zhang and coworkers proposed a one-pot, solvent-free synthesis of 2-anilinobenzoxazoles and 2-anilinobenzothiazoles **37** from anilines, CS_2_ and 2- aminophenol or 2-aminothiophenol using a sequential ball-milling protocol that combines the initial generation of an isothiocyanate (see Scheme 9 above) followed by its *in situ* reaction with the 2-amino(thio)phenol derivative.^40^

#### Imidazoles

The hydantoin (imidazolidine-2,4-dione) system is one of the imidazole derivatives with the highest pharmacological significance and is accessible, among other methods, by the classical Bucherer–Bergs reaction, a multicomponent process that was first reported in 1934. This transformation uses as starting materials aldehydes or ketones, potassium cyanide and ammonium carbonate, which acts as the source of two of the reaction components, namely ammonia and carbon dioxide.

**Scheme 26:**
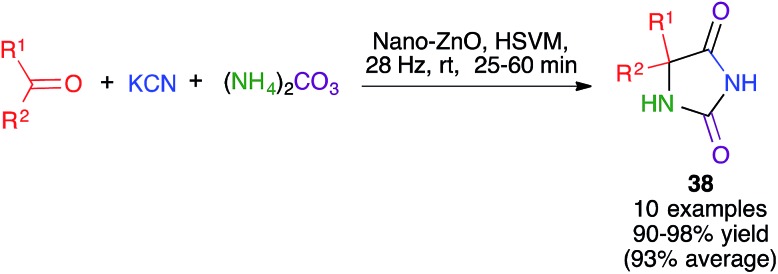
The mechanochemical Bucherer–Bergs reaction.

The Bucherer–Bergs reaction has been recently proved by Maddah to be amenable to mechanochemical methodology, affording hydantoins **38**. Following a thorough optimization process, a ZnO nanocatalyst was chosen and the best conditions involved high-speed vibration milling at 28 Hz in a stainless steel ball mill, although the number and composition of the balls was not specified (Scheme 26).^41^

**Scheme 27:**
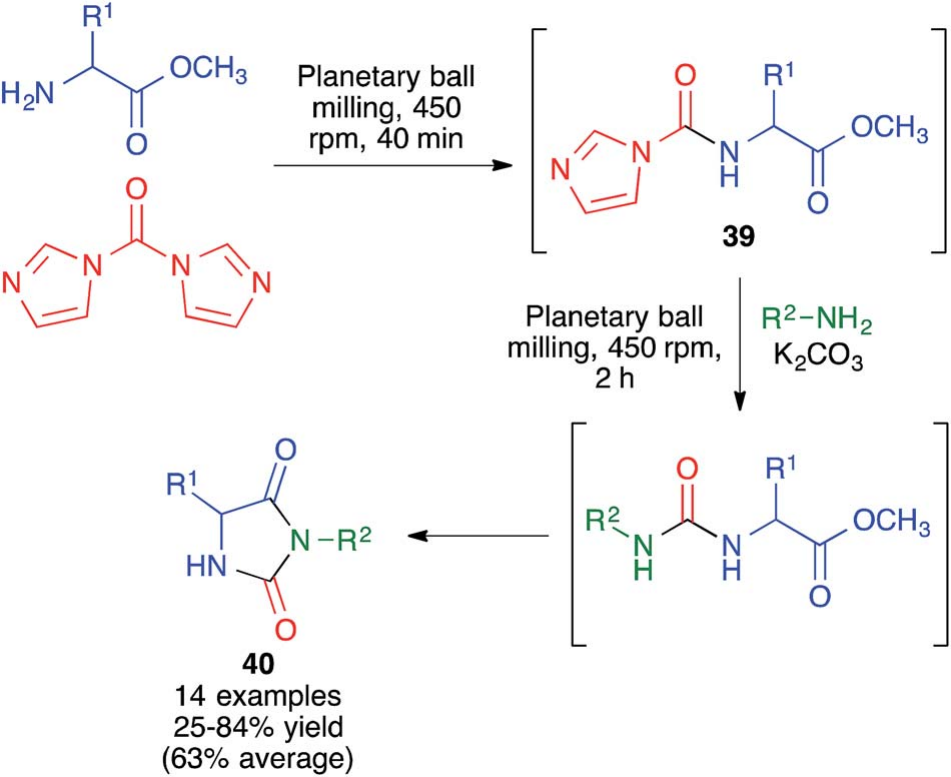
Mechanochemical synthesis of 3,5-disubstituted hydantoins.

In another approach, developed by Colacino and coworkers, 3,5-disubstituted hydantoins **40** were obtained by a sequential process that comprised the initial reaction between α-amino acids and carbonyldiimidazole under planetary ball milling conditions at 450 rpm using 50 stainless steel balls (5 mm, 5 mm diameter) for 40 min to generate intermediate **39**, followed by addition of a primary amine and potassium carbonate and additional 2 h ball milling under the same conditions (Scheme 27).^42^ Subsequent work by the same researchers proved that a liquid-assisted grinding protocol based on the addition of polyethylene glycols led to cleaner reaction profiles.^43^

**Scheme 28:**
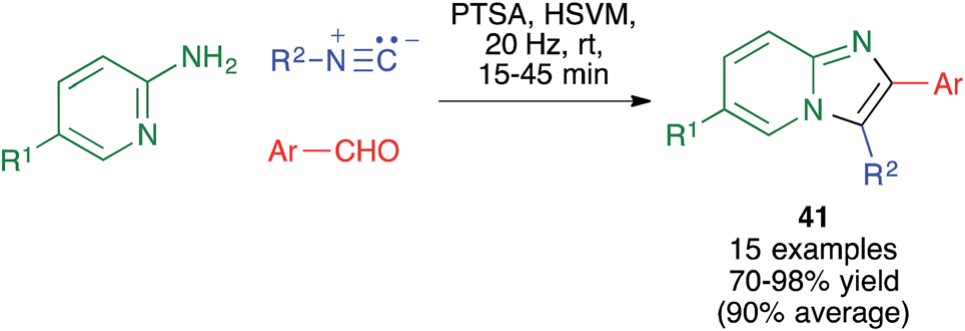
Three component synthesis of imidazo[1,2-*a*]pyridines using a mechanochemical Van Leusen-type reaction.

Maleki and coworkers have used a method related to the Van Leusen imidazole synthesis to obtain imidazo[1,2-*a*]pyridines **41** from 2-aminopyridines, aldehydes and isonitriles. The reaction was performed in a stainless steel ball mill at 20 MHz with two 12 mm diameter balls of the same material, using toluenesulfonic acid as the catalyst (Scheme 28).^44^

#### Triazoles

**Scheme 29:**
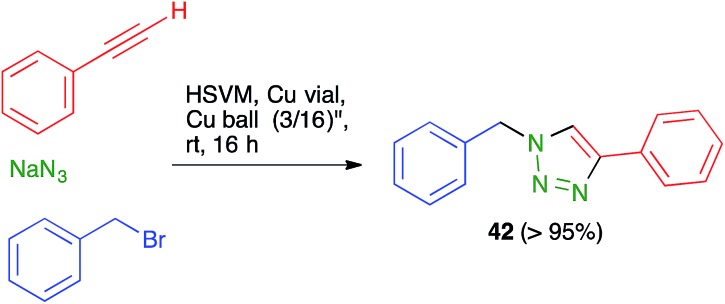
A three-component, mechanochemical click reaction.

The term “click chemistry” was introduced by Sharpless to describe reactions that are able to generate carbon–carbon and carbon-heteroatom bonds in high yield while being experimentally simple and affording crude mixtures that can be purified without chromatography. The first click reaction was the Copper-Catalyzed Azide-Alkyne Cycloaddition (CuAAC), a variation of the traditional Huisgens 1,3-dipolar cycloaddition that can be performed at room temperature in partially aqueous media. Solvent-free click reactions have been performed by milling together alkynes and azides using copper balls, which provide the catalyst by shedding copper particles besides transferring mechanical energy to the reactants. Mack and coworkers went one step further by performing the synthesis of the azide *in situ* and thus developing a multicomponent mechanochemical CuAAC reaction. A model reaction shown in Scheme 29 gave almost quantitative yield of compound **42**, but unfortunately the scope of the multicomponent method was not explored further. The authors state explicitly that they did not “observe any explosion or increased exothermicity due to milling azides”, in spite of initial concerns associated to the shock-sensitive nature of many alkyl azides. In fact, due to the fact that they are conducted in a sealed steel container, they came to regard these reactions as safer than those carried out in glassware.^45^

### 4.2. Six-membered heterocycles

#### Dihydropyridines

**Scheme 30:**
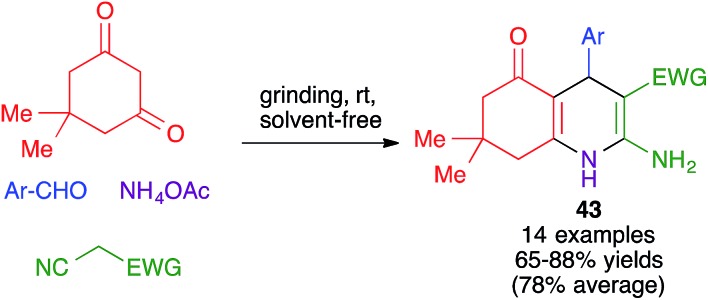
Application of the Hantzsch DHP synthesis to the preparation of polyhydroquinolines under mechanochemical conditions.

1,4-Dihydropyridines (DHPs) are an important family of antagonists of Ca^2+^ channels, with widespread clinical application as vasodilators and having also promising neuroprotective properties.^46^ The multicomponent reaction between aromatic aldehydes, β-dicarbonyl compounds (two molecules) and ammonia, known as the Hantzsch dihydropyridine synthesis, is the earliest and best-known method for the preparation of this skeleton.^47^ By using cyclic 1,3-cyclohexanediones as one of the β-dicarbonyl components, the reaction can be easily adapted to the synthesis of polyhydroquinolines. A mechanochemical version of the latter reaction was developed by Hundal *et al.* and involved grinding mixtures of aldehydes, dimedone, acyclic active methylene compounds and ammonium acetate at room temperature in a mortar in the absence of solvent (Scheme 30).^48^ The reaction gave good to excellent yields of compounds **43** when aromatic aldehydes were employed, tolerating the use of starting materials with electron-rich and electron-deficient rings as well as heterocyclic aldehydes, but gave poor results with aliphatic aldehydes. Interestingly, the authors proved that the reaction could be performed at a 100 mmol scale with no loss in yield.

**Scheme 31:**
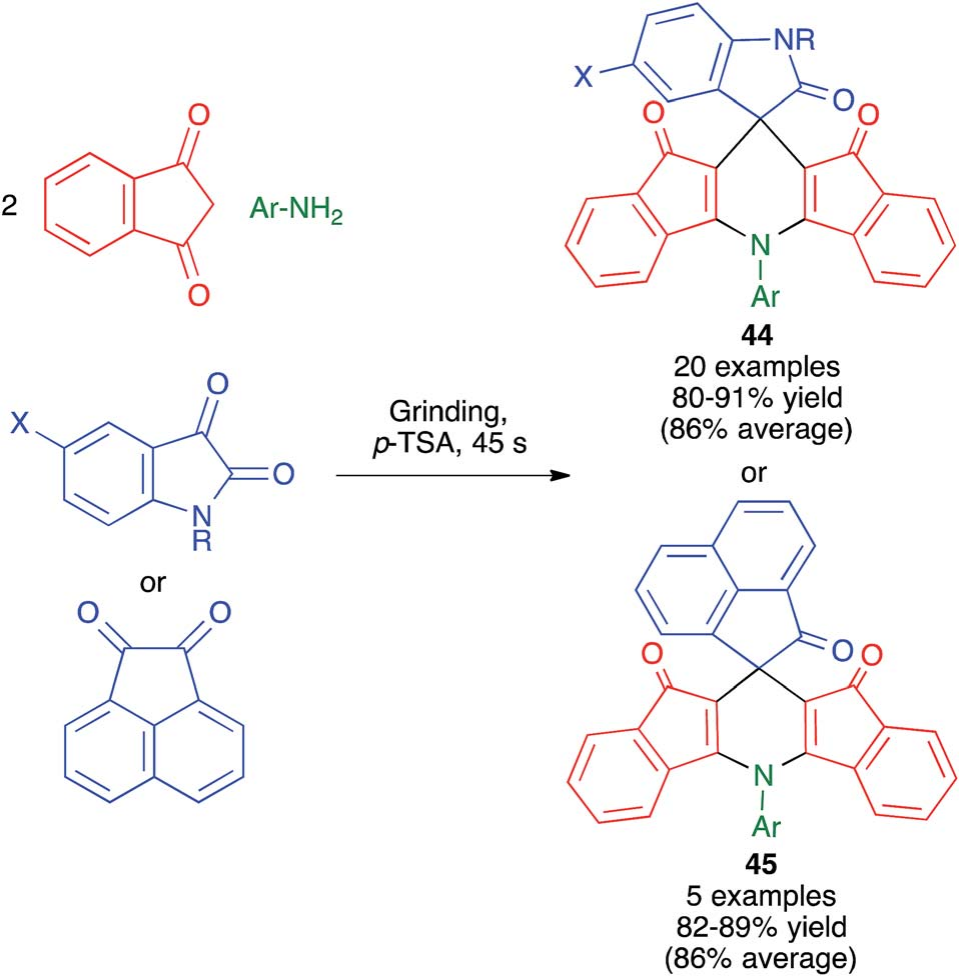
Synthesis of spiro compounds under manual grinding conditions.

Replacement of the aldehyde component by a sufficiently reactive cyclic ketone allows the preparation of spiro compounds by Hantzsch-like chemistry. As shown in Scheme 31, Bazgir and coworkers have described a one-pot four-component synthesis of complex spiro ring systems **44** and **45***via* the reaction of 1,3-indanedione with aromatic amines and isatin or acenaphthylene-1,2-dione under manual grinding in solvent-free conditions and in the presence of *p*-toluenesulfonic acid as a catalyst.^49^

**Scheme 32:**
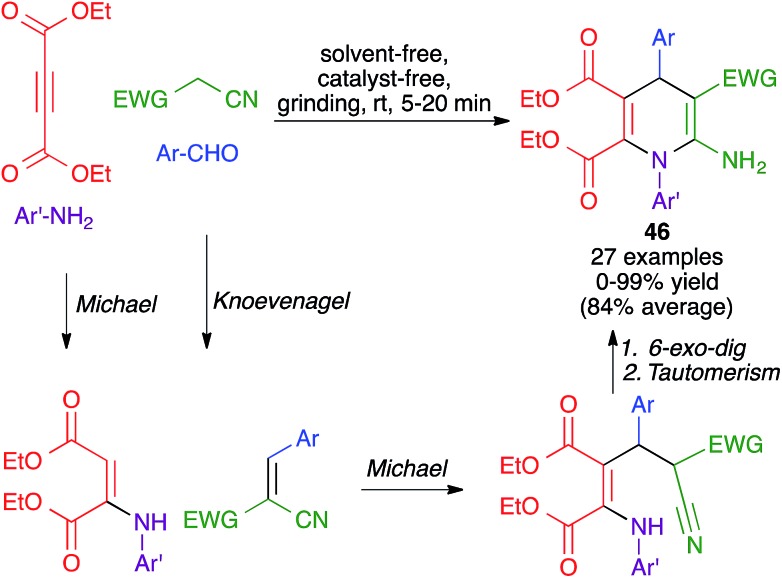
Mechanochemical four-component synthesis of DHPs from an acetylenedicarboxylate.

Another mechanochemical multicomponent reaction that leads to the formation of dihydropyridine rings was developed by Kamur and Sharma and was achieved by grinding together aldehydes, amines, diethyl acetylenedicarboxylate and malononitrile or ethyl cyanoacetate, in a porcelain mortar for 5–20 minutes, to furnish compounds **46**. Its mechanism was proposed to comprise an initial Michael reaction between diethyl acetylenedicarboxylate and the aniline, concomitant with a Knoevenagel reaction between the aldehyde and the active methylene compound. The formation of the final product would be completed by the combination of a Michael addition between the two fragments, a 6-*exo*-dig cyclization and imine-enamine tautomerism (Scheme 32).^50^

**Scheme 33:**
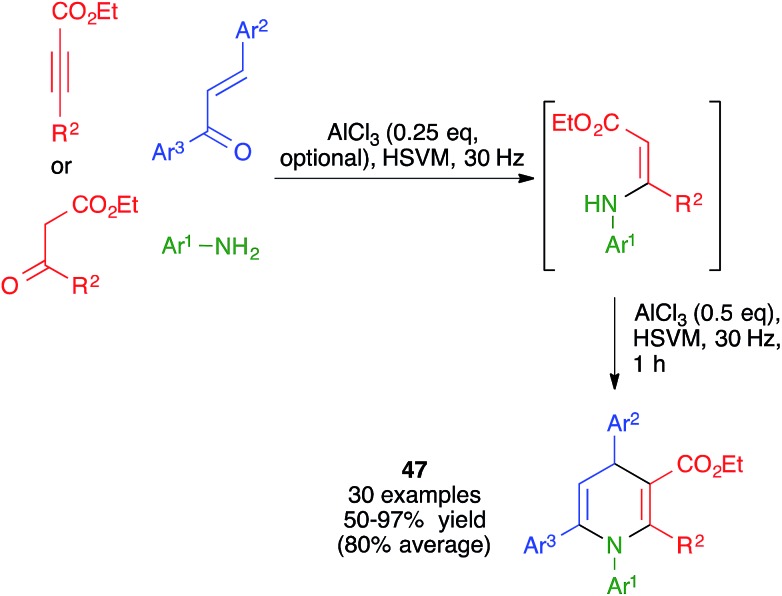
Mechanochemical four-component synthesis of DHPs from chalcones.

The mechanochemical reaction between β-enaminones and chalcones in the presence of AlCl_3_ was studied by Zhang and coworkers under ball milling, using an experimental protocol that involved the *in situ* generation of the enaminones by Michael addition of anilines to acetylene dicarboxylates or, in some cases, by their condensation with β-ketoesters. These researchers found that, in contrast with a previously described similar reaction that had been performed under thermal conditions, the mechanochemical protocol afforded 1,4,6-triaryl-1,4-dihydropyridine derivatives **47** (Scheme 33).^51^

**Scheme 34:**
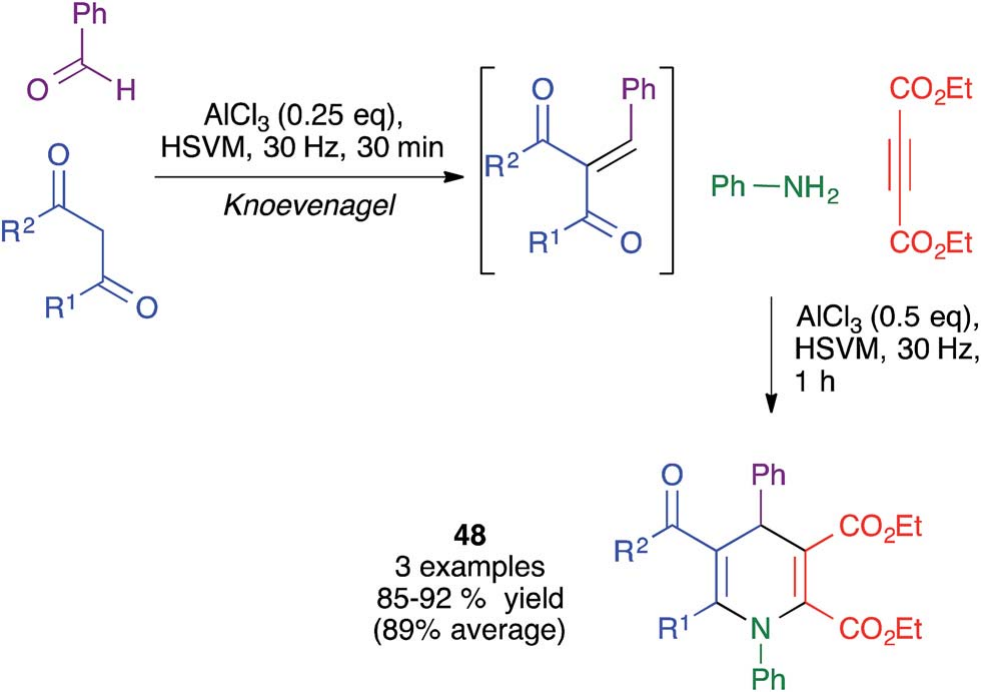
Knoevenagel-initiated mechanochemical four-component synthesis of DHPs.

The same authors also showed the feasibility of performing a similar transformation from *in situ*-generated Knoevenagel adducts to yield dihydropyridines **48**, albeit in a very limited number of cases. Interestingly, they found that, when starting from 1,3-cyclohexanedione derivatives, the same conditions afforded fused pyran derivatives rather than the expected fused dihydripyridines (Scheme 34).^51^

#### Pyrimidines

**Scheme 35:**
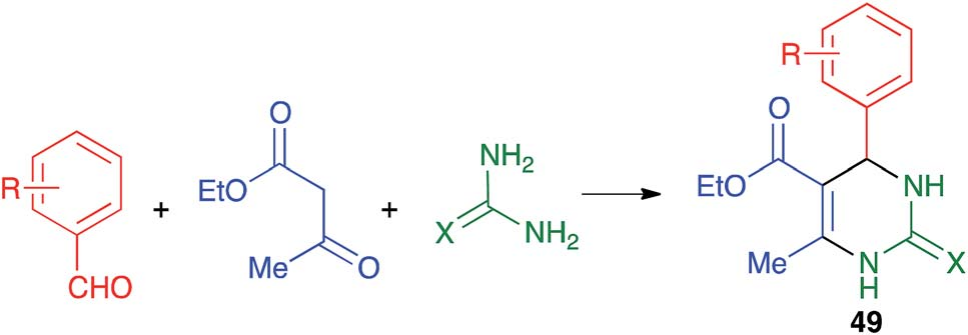
The Biginelli reaction.

The Biginelli reaction from ethyl acetoacetate, aromatic aldehydes and urea derivatives to yield 3,4-dihydropyrimidin-2(1*H*)-one derivatives **49** (Scheme 35) is one of the three-component reactions discovered in the 19^th^ century. It has been well studied under mechanochemical conditions, compared to other multicomponent transformations, using both manual grinding and ball-milling approaches.

Manhas *et al.* studied the reaction by manually grinding the starting materials and *p*-TSA, a Brønsted acid, and examined solid/solid, solid/liquid and liquid/liquid combinations. They found that to improve the grinding process it was useful to add crystalline MgSO_4_·7H_2_O to the reaction mixture. In some cases where water-soluble final products were obtained, they replaced this grinding auxiliary by sand.^52^ Alternatively, Singh and co-workers have proposed SnCl_4_·5H_2_O as a Lewis acid catalyst for the “grindstone” Biginelli reaction.^53^

A number of ball-milling studies have also been performed, focused on the planetary milling approach. In these experiments, the ball weight/reagent weight is an important parameter that was determined by M'hamed to have an optimal value between 5 and 8 for the solvent- and catalyst-free Biginelli reaction.^54^,^55^

**Scheme 36:**
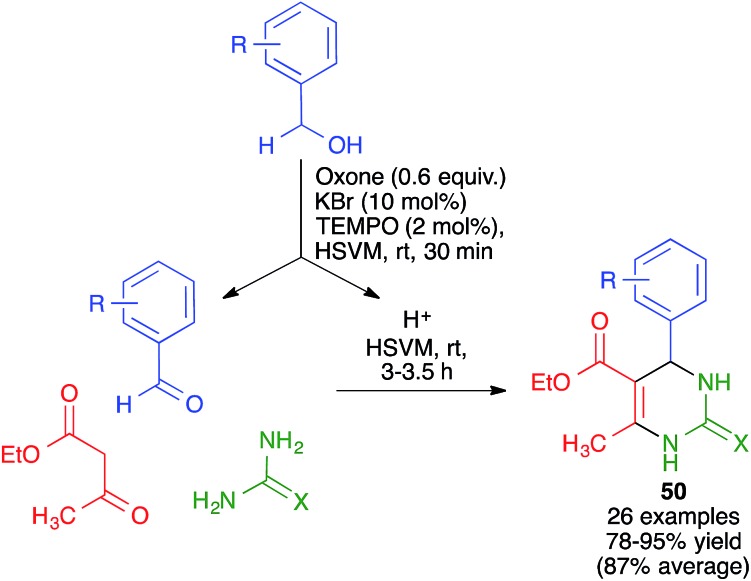
Mechanochemical Biginelli reactions with *in situ* preparation of the aldehyde component.

Mal and coworkers have studied the coupling of the Biginelli reaction with the *in situ* preparation of the catalyst and one of the starting materials. Thus, they found that bromonium-catalyzed oxidation of benzyl alcohols, achieved by their treatment with mixture of KBr, oxone and TEMPO, followed by addition to the same pot of ethyl acetoacetate and urea or thiourea, afforded pyrimidines **50** in good to excellent yields (Scheme 36). The proton liberated from the starting material upon its oxidation was assumed to act as a catalyst.^56^ The whole procedure was performed in a stainless steel milling jar containing a single stainless steel ball (5 mm diameter), but, interestingly, the reaction did not work at all in solution.

Finally, Jang *et al.* demonstrated the extension of the mechanochemical Biginelli reaction to the synthesis of fused pyrimidines by employing as starting materials β-dicarbonyl compounds, aldehydes and 1*H*-benzo[*d*]imidazol-2-amine or benzo[*d*]thiazol-2-amine in the presence of 0,4% of ZnO nanoparticles as catalyst.^57^

#### Quinoline derivatives

The Povarov reaction, *i.e.*, the [4 + 2] cycloaddition between imines and electron-rich olefins, is a well-known method for the synthesis of 1,2,3,4-tetrahydroquinolines. Zhang and coworkers have reported the use of the inexpensive, easily available and non-toxic FeCl_3_ as an efficient catalyst for the mechanochemical reaction between *in situ*-generated *N*-aryl aldimines and styrene to furnish *cis*-2,4-diphenyl-1,2,3,4-tetrahydroquinoline derivatives **51** with complete diastereoselectivity. This feature is uncommon in Povarov reactions carried out from acyclic olefins, and was explained by the high local concentration of the reactants under the solvent-free conditions, which may result in an enhanced second-order reaction rate and thus a higher preference for the selective formation of the kinetic product in comparison with the solution-phase reaction.^58^

**Scheme 37:**
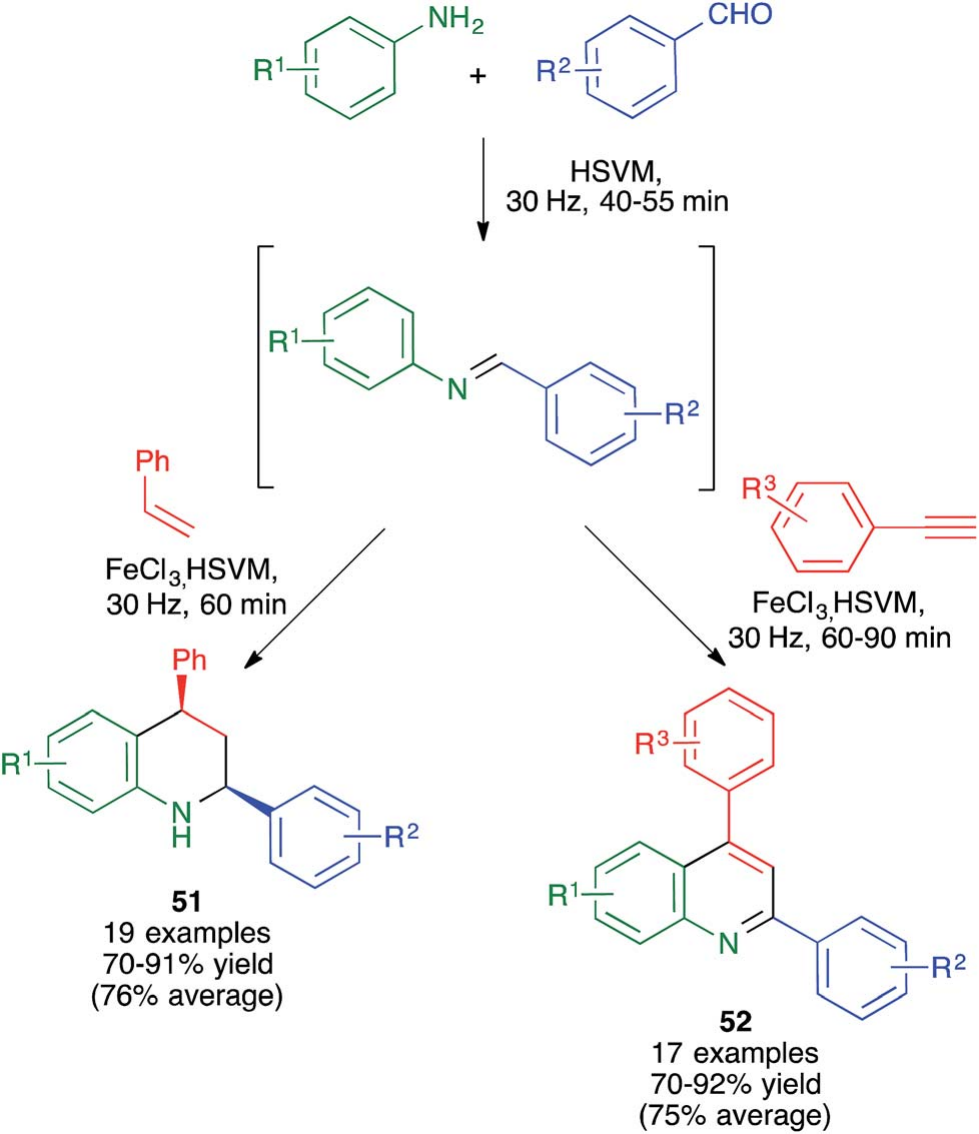
The mechanochemical Povarov reaction.

The same group reported later a similar transformation starting from phenylacetylene derivatives and affording fully aromatic quinolines **52** (Scheme 37).^59^

#### Pyran and fused pyran derivatives

**Scheme 38:**
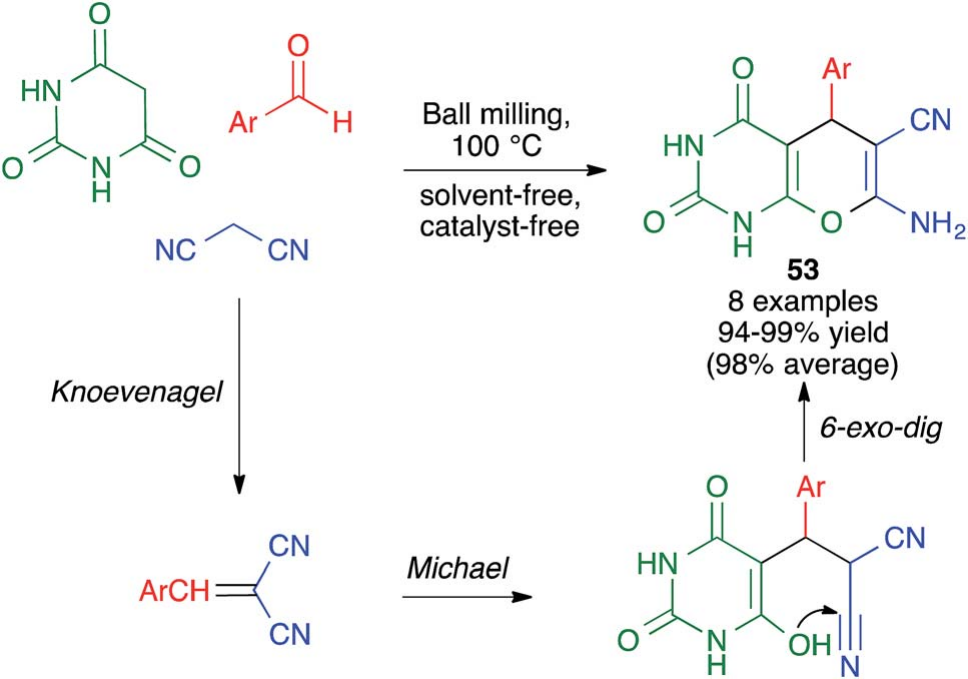
Solvent- and catalyst-free synthesis of pyrano[2,3-*d*]pyrimidine-2,4-diones.

Naimi-Jamal and coworkers described a solvent-free and catalyst-free synthesis of pyrano[2,3-*d*]pyrimidine-2,4(1*H*,3*H*)-diones **53** from aromatic aldehydes, malononitrile and barbituric acid. This was one of the very first reports of a multicomponent reaction carried out under ball milling conditions. However, its energy source was not purely mechanical since the authors employed a modified ball mill with boiling water as circulant that was used to heat the reaction. This transformation allowed the presence of either electron-withdrawing or electron-donating groups in the aldehydes, with excellent isolated yields, although the number of examples was rather limited and was proposed to take place by a Knoevenagel/Michael/6-*exo*-dig cyclization sequence (Scheme 38).^60^

**Scheme 39:**
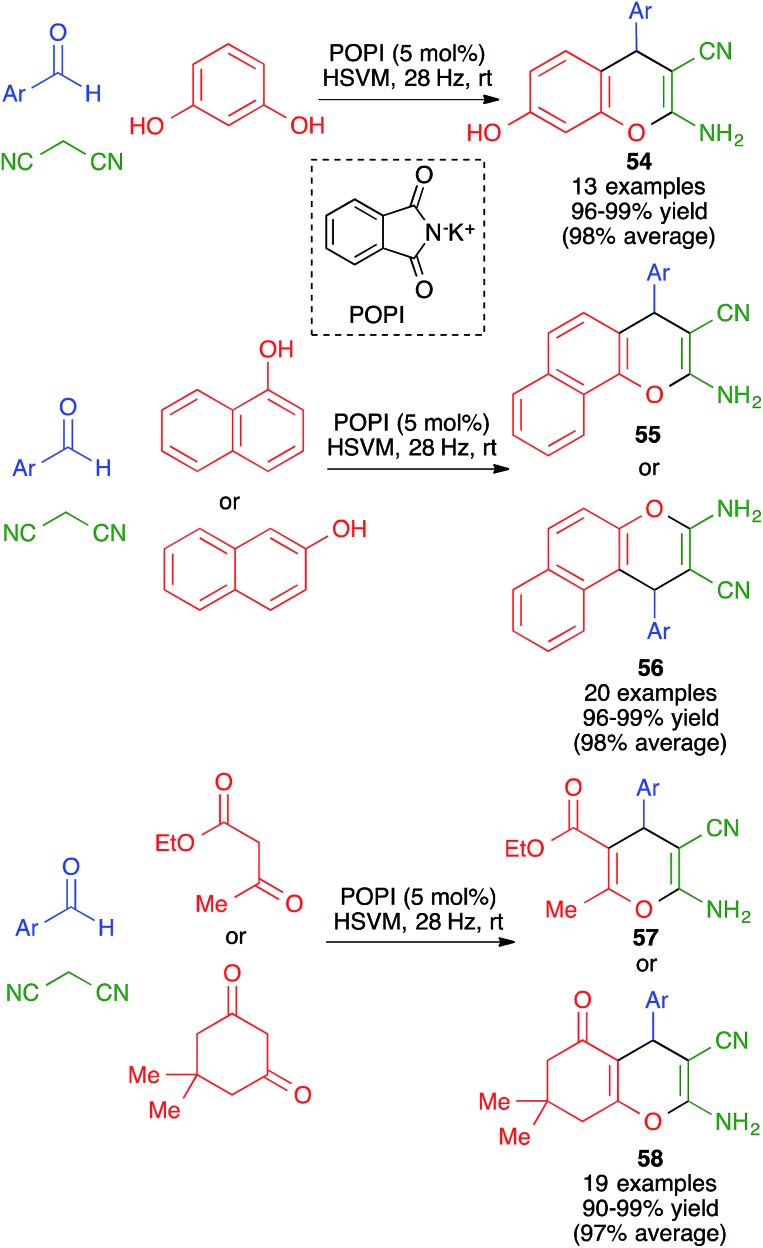
Potassium phthalimide (POPI) as an organocatalyst for the mechanochemical synthesis of fused 4*H*-pyran derivatives.

In closely related work (Scheme 39), Eslami *et al.* have developed a one-pot three-component synthesis of 2-amino-3-cyano-4*H*-pyran or annulated pyran derivatives (compounds **54–58**) from aromatic aldehydes, malononitrile and enolizable C–H activated acidic compounds (including phenols) under purely mechanochemical conditions (2 stainless steel balls, 28 Hz) at room temperature. In all cases, the reaction was promoted by the mildly basic organocatalyst potassium phthalimide (POPI).^61^

In an alternative mechanochemical approach, Crawford and James have described the preparation of compounds **58** using the twin screw extrusion (TSE) technique, in the absence of solvent.^8^

**Scheme 40:**
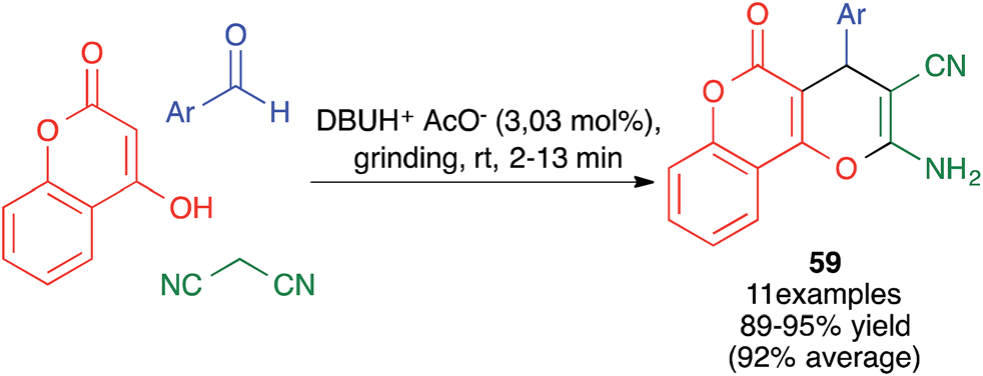
Synthesis of dihydropyrano[*c*]chromenes by manual grinding.

**Scheme 41:**
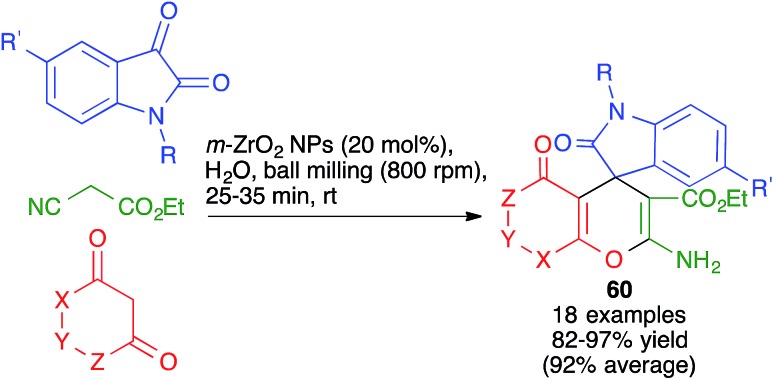
Synthesis of spiro compounds **60** under planetary ball milling.

Other variations of this chemistry that involve modifications in the carbonyl electrophile have been developed. One of them was disclosed by Raval and coworkers and allows the very efficient synthesis of 3,4-dihydropyrano[*c*]chromenes **59** from 4-hydroxycoumarin, acting as the β-dicarbonyl component, in the presence of the ionic liquid DBUH^+^·AcO^–^, under manual grinding conditions (Scheme 40).^62^ In work reported by Bajpai *et al.*, the preparation of complex spiro systems **60** from isatins, in the presence of monoclinic zirconia nanoparticles and using a planetary ball mill that contained 16 Al_2_O_3_ balls (10 mm in diameter) and operated at 800 rpm was described (Scheme 41).^63^

**Scheme 42:**
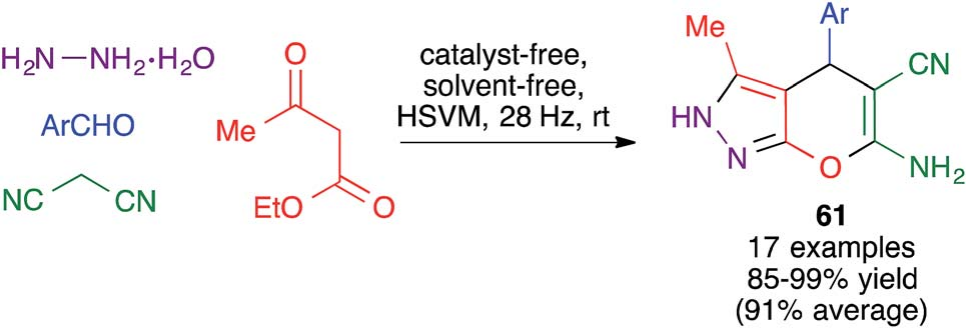
Mechanochemical synthesis of pyrano[2,3-*c*]pyrazoles.

Related reactions involving modifications of the β-dicarbonyl component were also successfully implemented. Thus, Dekamin and coworkers showed that a pyrazolone derivative, generated *in situ* from hydrazine hydrate and ethyl acetoacetate, reacted with aromatic aldehydes and malononitrile under solvent-free mechanochemical conditions to furnish 2,4-dihydropyrano[2,3-*c*]pyrazoles **61** (Scheme 42). The optimized experimental conditions involved the use of high-speed milling vibration with two stainless steel balls at 28 Hz, in the absence of any solvent or catalyst.^64^

**Scheme 43:**
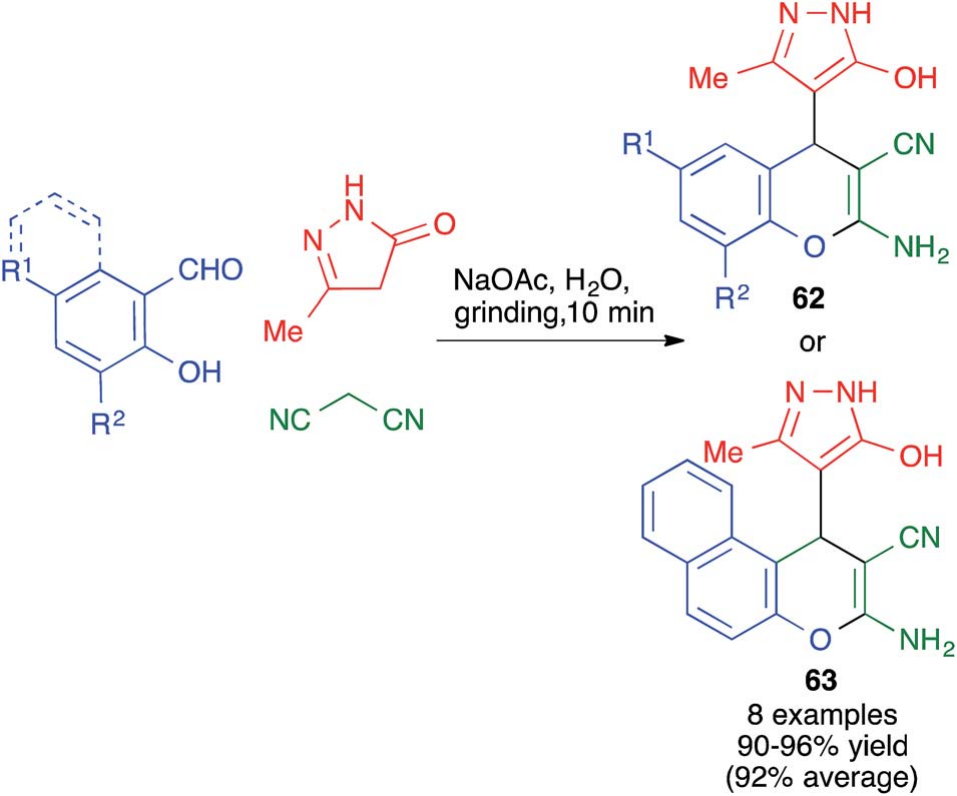
Mechanochemical synthesis of pyrans from aldehydes bearing a *o*-hydroxy group.

**Scheme 44:**
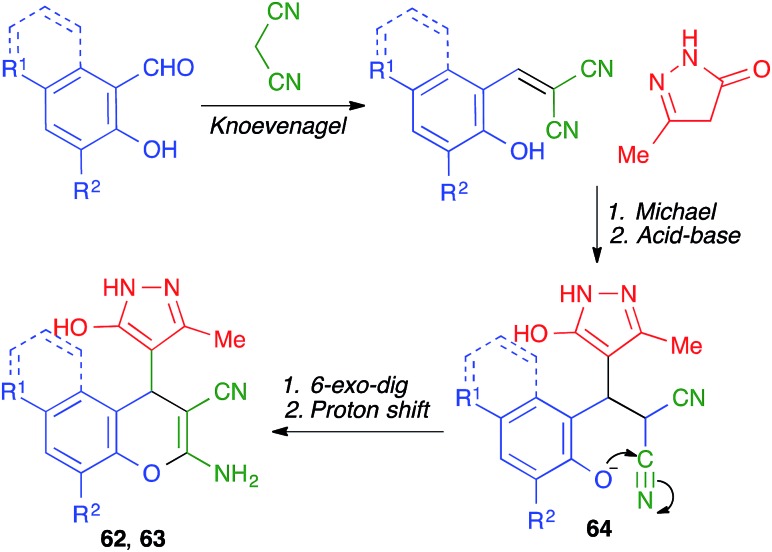
Mechanistic proposal that accounts for the formation of **45** and **46**.

A different mode of cyclization from very similar starting materials was achieved by introducing an *o*-hydroxy group in the starting aldehyde. Thus, Elinson and coworkers performed the reaction between salicylaldehydes, pyrazolones and malononitrile by manually grinding the starting materials in a mortar in the presence of small quantities of water and 10 mol% of sodium acetate as a mild base. Instead of leading to cyclization from the pyrazolone oxygen, as in the previous case, this reaction afforded compounds **62** or **63**, where the aromatic ring of the aldehyde is fused to the pyrane ring rather than attached to the C-4 position (Scheme 43).^65^ The mechanism proposed to explain this transformation starts with a base-promoted Knoevenagel–Michael sequence followed by interception of the *o*-hydroxy substituent by one of the nitrile groups in intermediate **64** (Scheme 44).

#### BODIPY dyes

**Scheme 45:**
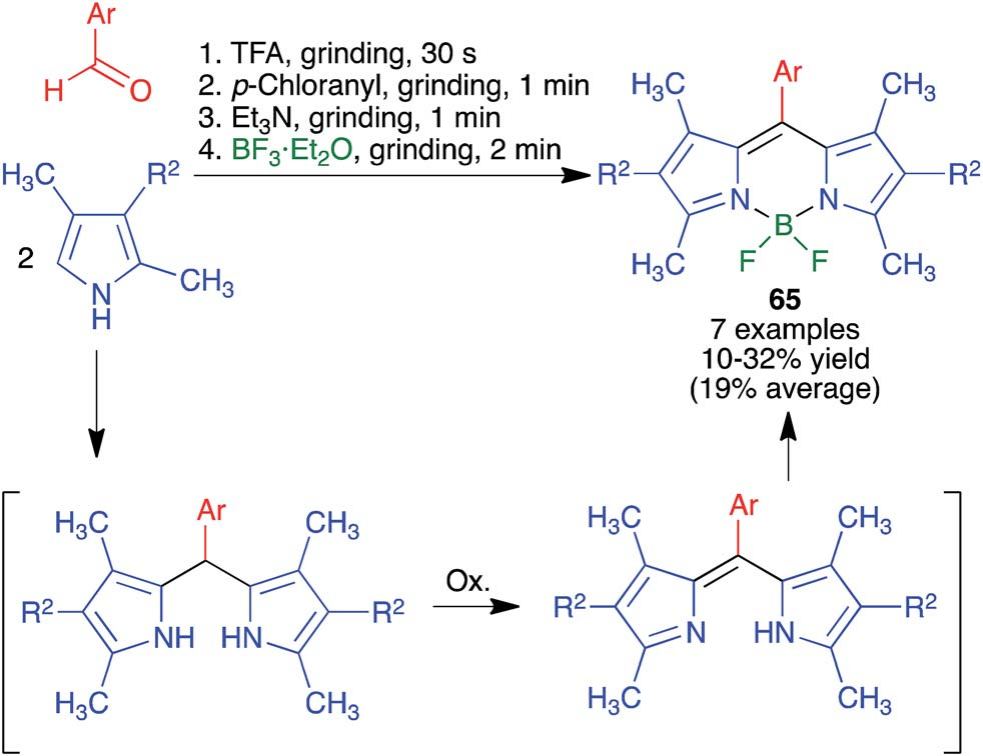
Mechanochemical synthesis of BODIPY dyes under manual grinding using a mortar and pestle.

BODIPY dyes are organic luminophores that have high quantum yields and tunable fluorescent properties, and have attracted much attention in recent years for a variety of applications. Dzyuba has shown that compounds **65** can be obtained under essentially solvent-free conditions using grinding with a pestle and mortar (Scheme 45). The yields were poor but comparable to those obtained in solution, and the mechanochemical protocol had the advantage of requiring very short reaction times (5 min *vs.* several hours to days).^66^

## 5. Mechanochemical synthesis of coordination and organometallic compounds

**Scheme 46:**
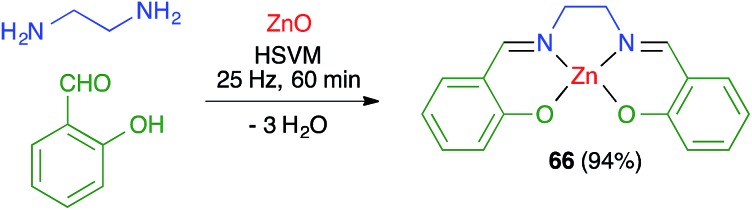
Mechanochemical synthesis of a zinc–salen complex.

**Scheme 47:**
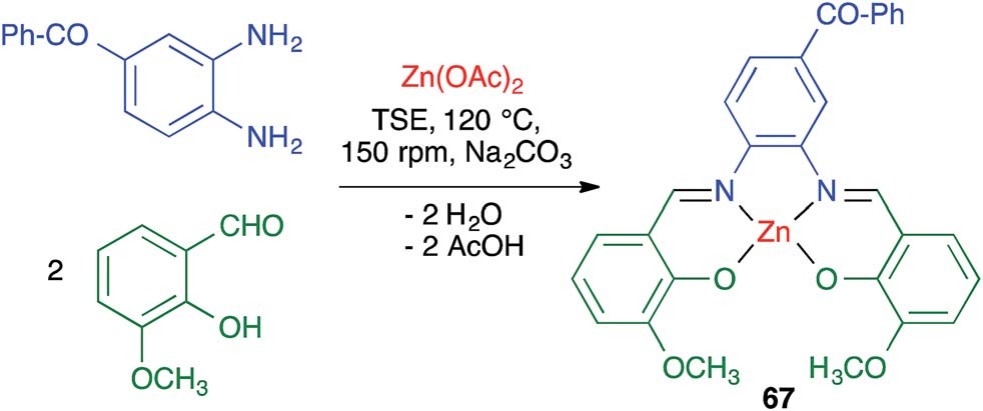
Preparation of a salicylaldehyde imine complex under twin screw extrusion.

James and coworkers described the three-component synthesis of salen complexes under high-speed vibration milling in a shaker mill (Scheme 46).^67^ In the initial experiments, the required salen ligands were obtained from ethylenediamine and the suitable salicylaldehyde derivative by ball milling (30 min, 25 Hz) in the absence of any solvent or catalyst, and this was followed by addition of a cation salt and additional ball milling under the same conditions and using methanol as an auxiliary for liquid-assisted grinding. As a subsequent development, the authors proved that the Zn(ii) complex **66** could be obtained in a single operation. Furthermore, Crawford and James showed that closely related Zn complexes **67**, derived from salicylaldehyde and *o*-phenylenediamine derivatives, were readily accessible by application of the twin screw extrusion (TSE) technique (Scheme 47).^8^

**Scheme 48:**
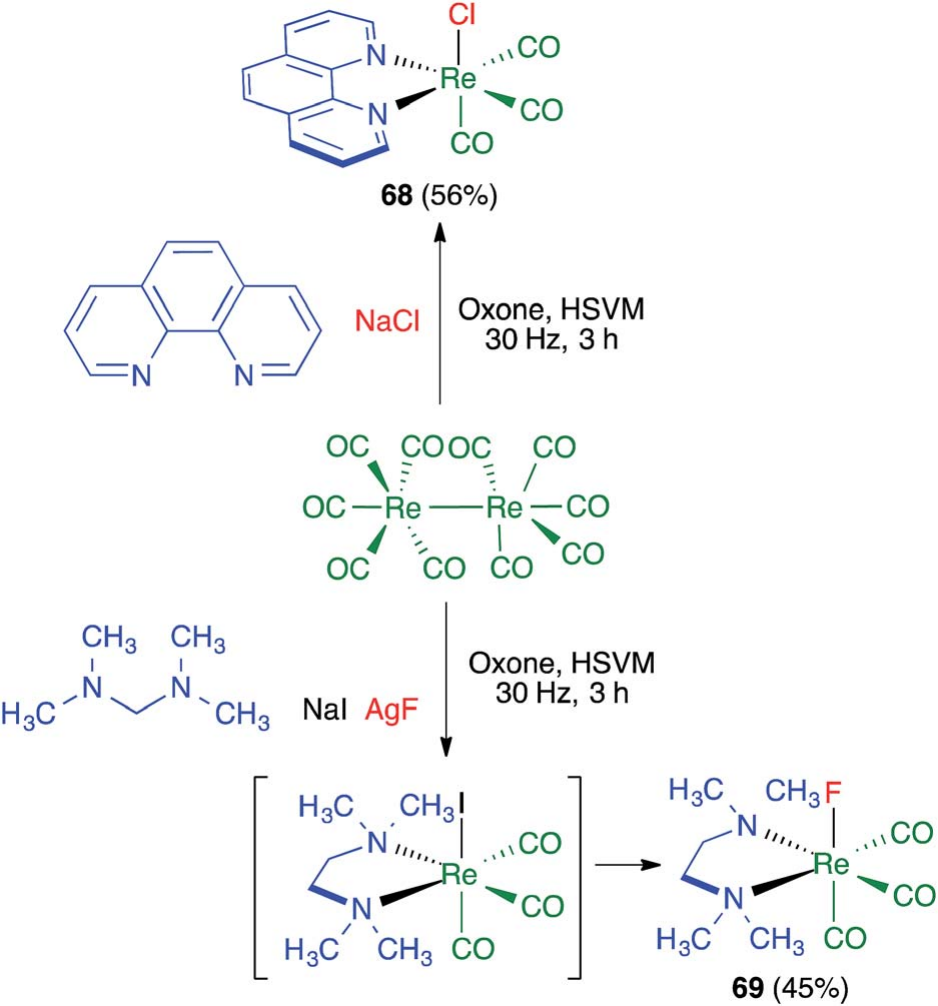
Mechanochemical synthesis of Re complexes.

Friščić and coworkers demonstrated for the first time the combination of redox and ligand exchange reactions in a multicomponent process.^68^ Thus, rhenium complexes **68** were obtained from Re_2_(CO)_10_, 4,5-phenanthroline and sodium chloride in the presence of oxone, under high-speed vibration milling conditions. Complex **69**, derived from *N*,*N*,*N*′,*N*′-tetramethyl-ethylenediamine and containing fluoride as a ligand, were obtained similarly, although with an *in situ* iodide–fluoride exchange step (Scheme 48).

**Scheme 49:**
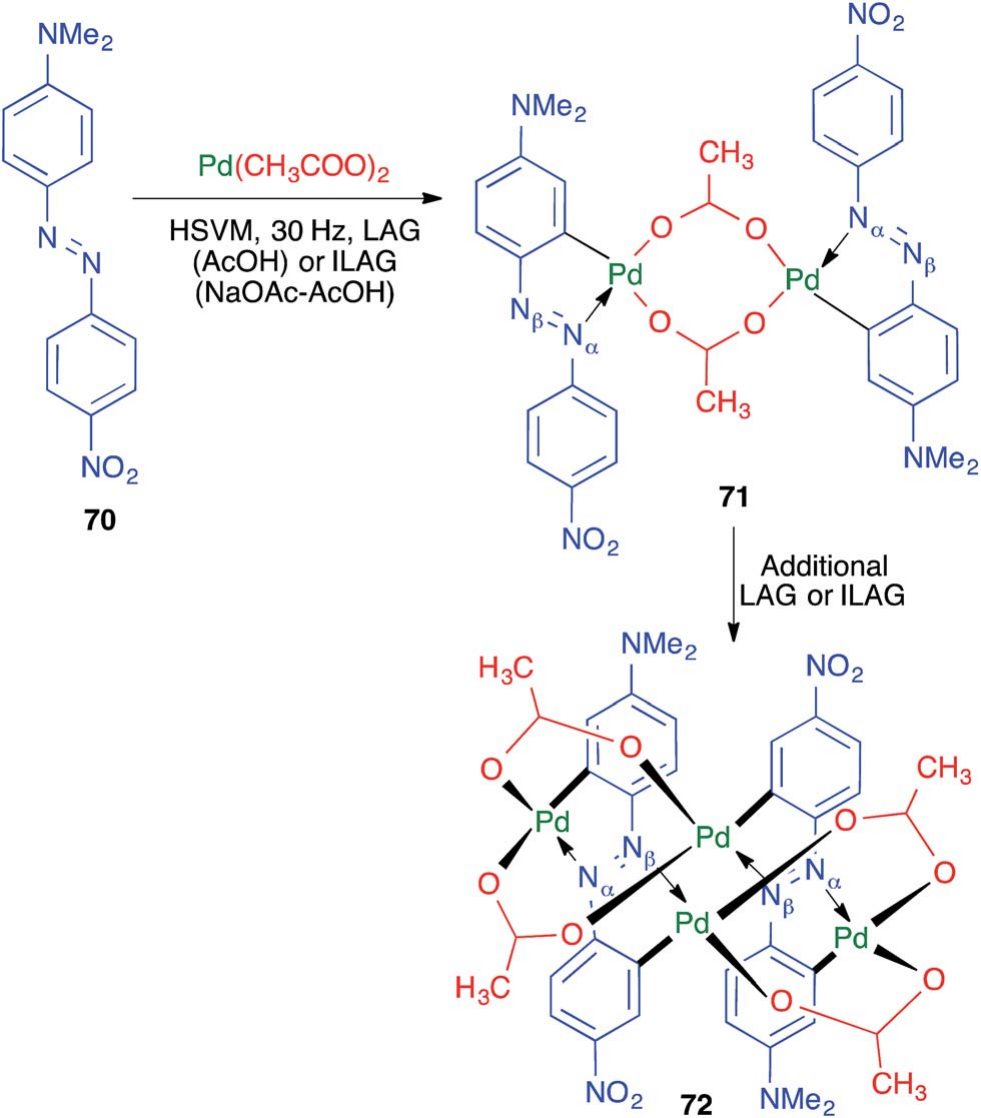
Mechanochemical synthesis of a Pd metallosupramolecular complex.

The first example of a direct transition-metal-mediated mechanochemical activation of aromatic C–H bonds was achieved by Ćurić and coworkers, resulting in cyclopalladated complexes **72** from azo derivative **70**.^69^ The palladation reaction was performed under liquid-assisted grinding conditions (HSVM at 30 Hz, using a single stainless steel ball 10 mm in diameter) in the presence of glacial acetic acid and led initially to **71**; thus, the palladation was found to be fully regioselective in favor of the aromatic ring of **70** containing the dimethylamino group (Scheme 49). Additional grinding afforded **72**, from a new palladation reaction, and both steps could be performed in a one-pot operation, as shown by monitoring the reaction by *in situ* solid-state Raman spectroscopy. The authors found that the initial palladation that furnishes **71** could be carried out in solution, although it required prolonged reaction times, but the second reaction, leading to compound **72**, could only be performed in the solvent-free mechanochemical conditions.

## 6. Multicomponent supramolecular chemistry under mechanochemical conditions

Rotaxanes are one of the main types of supramolecular mechanically interlocked systems, and are of interest as potential actuators and switches in molecular devices. They contain a linear molecule threaded through a macrocycle, with the threading unit having bulky moieties (stoppers) at both ends, thereby preventing the dissociation (dethreading) of the whole structure. The synthesis of rotaxanes is challenging and benefits from the use of solid-state conditions, since solution chemistry often leads to side products during the stoppering stage.

**Scheme 50:**
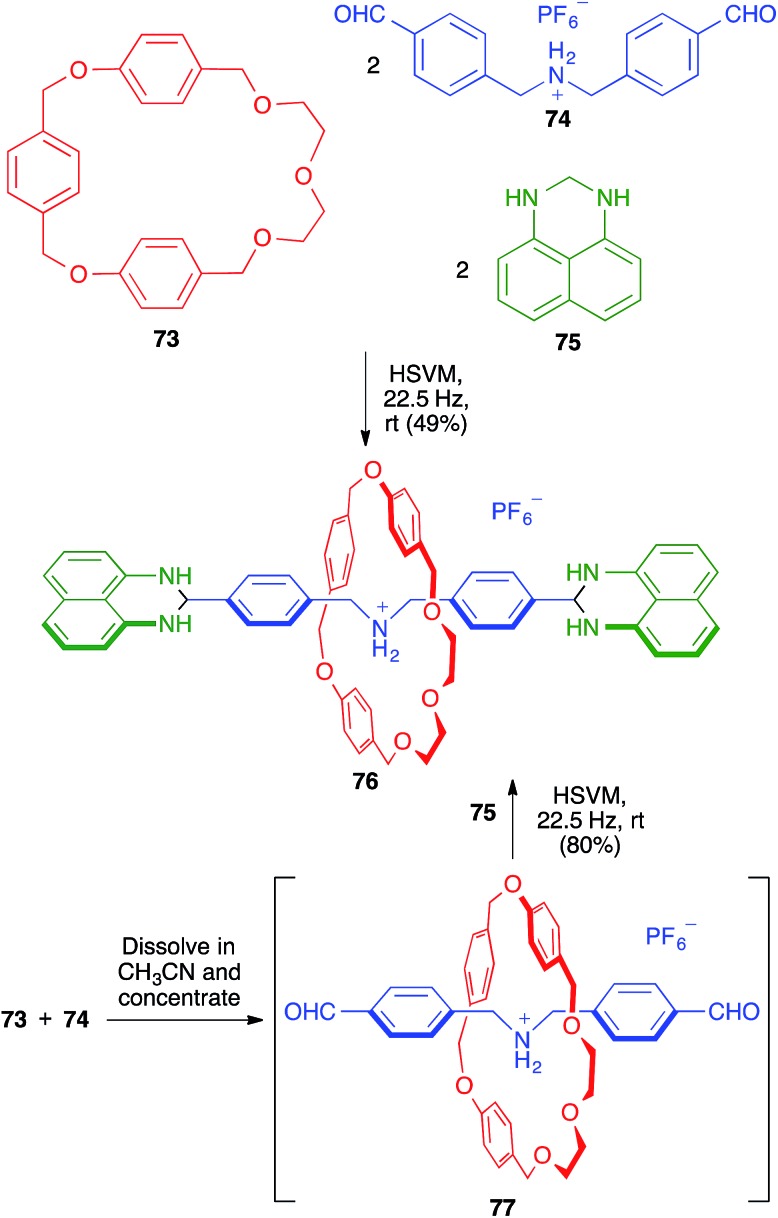
Mechanochemical multicomponent synthesis of [2]-rotaxanes.

In this context, the one-pot synthesis of [2]rotaxane **76** developed by Chiu and coworkers starting from the macrocyclic (**73**), threadlike (**74**) and stoppering (**75**) components can be considered a remarkable achievement. The reaction was carried out in the absence of solvent, in a mixer stainless steel mill containing two stainless steel balls (7 mm in diameter). The same process could be performed more efficiently in a sequential fashion by first mixing **73** and **74** in acetonitrile, followed by evaporation to generate *in situ* the pseudo-rotaxane **77**, which was then reacted with **75** under the previously mentioned HSVM conditions (Scheme 50).^70^ The same strategy allowed the efficient preparation of [4]rotaxanes.

**Scheme 51:**
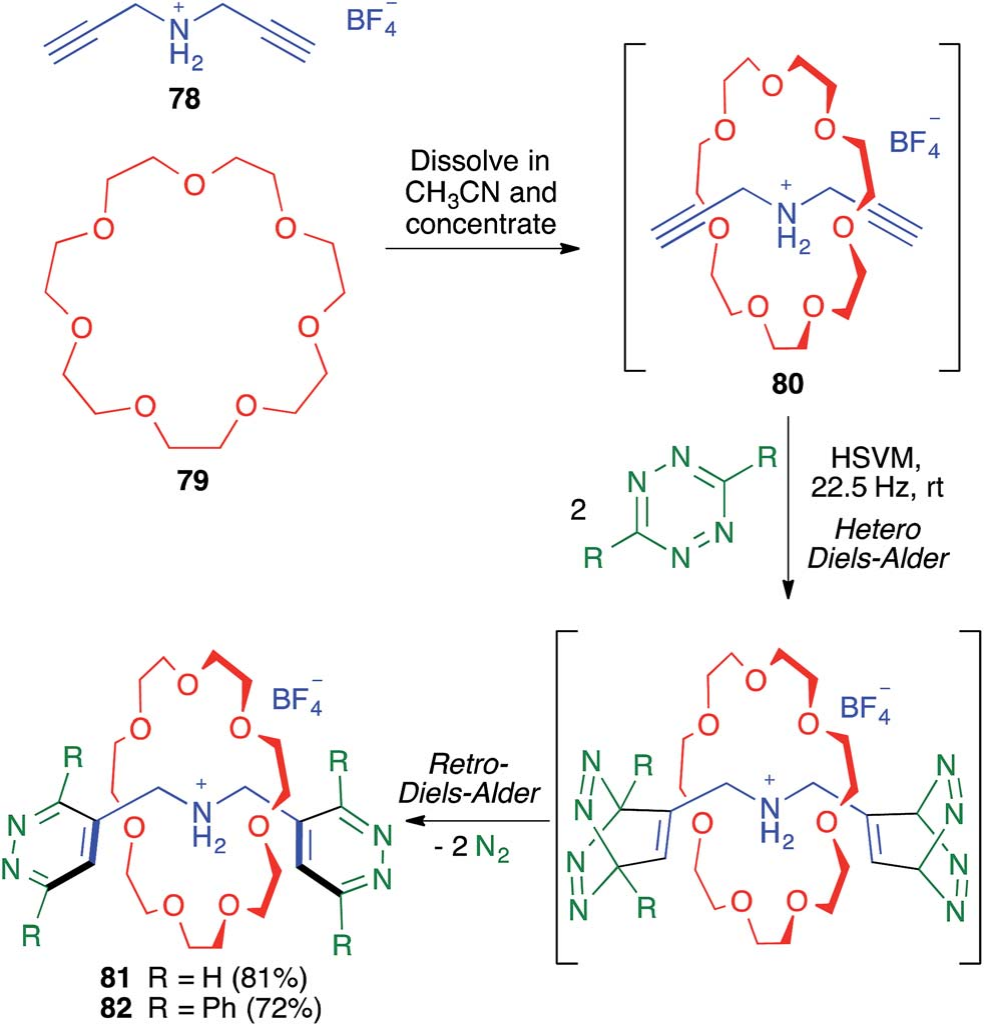
Synthesis of small [2]-rotaxanes using a sequential multicomponent reaction comprising mechanochemical steps.

As shown in Scheme 51, the Chiu group demonstrated the flexibility of their multicomponent approach through the preparation of the smallest rotaxanes known at that time (compounds **81** and **82**). To this end, they employed as the thread-like component the bis-propargylamine **78**, which afforded the pseudo-rotaxane **80** by its dissolution in acetonitrile, together with crown ether **79**, followed by solvent evaporation. The terminal alkynes were then used to construct the two 1,2-diazine stoppering fragments by a mechanochemical double hetero Diels–Alder reaction with a tetrazine derivative followed by an *in situ* double retro Diels–Alder with extrusion of two molecules of nitrogen.^71^,^72^

**Scheme 52:**
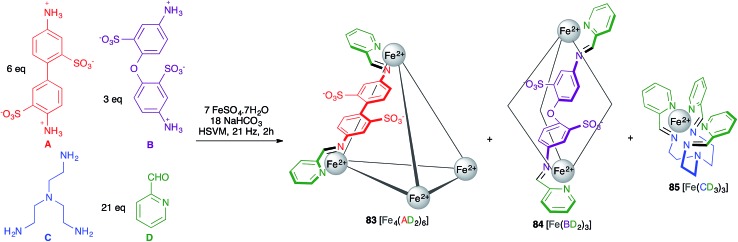
Mechanochemical synthesis of metallosupramolecular complexes. For clarity, in compounds **81** and **82** only one of the six AD or three BD chelating units is shown.

Rissanen and Mal described an example of self-sorting of three different Fe(ii) complexes. Thus, the starting materials **A**, **B**, **C** and **D** shown in Scheme 52 were mixed with a Fe^2+^ salt under HSVM and afforded mixtures of three complexes with broadly divergent architectures, namely the cage compound **83** and the helicates **84** and **85**.^73^ By adding additional subcomponent **B**, **83** was quantitatively transformed into **84**, which in turn became **85** upon addition of component **C**, this behaviour reflecting the thermodynamic stabilities of the three structures.

## 7. Synthesis of organic materials by mechanochemical multicomponent reactions

### 7.1. Nanocarbon materials

Fullerenes, carbon nanotubes and graphite have low solubilities in common organic solvents and water. For this reason, the chemical functionalization of these materials is challenging and solvent-free approaches, including mechanochemical ones, are very attractive.^74^ Nevertheless, only a few multicomponent reactions have been performed on fullerene substrates under mechanochemical conditions.

**Scheme 53:**
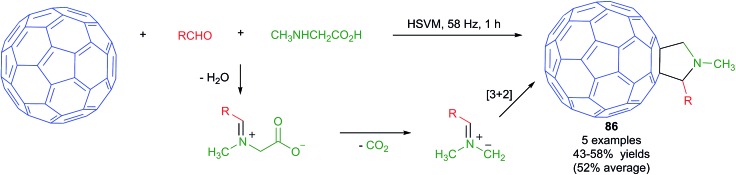
Synthesis of functionalized fullerenes *via* a mechanochemical Prato reaction.

The Prato reaction is a particular case of the well-known 1,3-dipolar cycloaddition of azomethine ylides to olefins applied to the functionalization of fullerenes and nanotubes. In this context, the mechanochemical reaction of fullerenes with the dipolar species arising from an *in situ* condensation/decarboxylation process from *N*-alkylglycine derivatives and aldehydes (Scheme 53) was found to provide fulleropyrrolidines **86** in moderate yields. The reactions were performed in a vibrating mill using a stainless steel ball and a vibration frequency of 58 Hz, and was the first example of a Prato reaction performed under HSVM conditions.

**Scheme 54:**
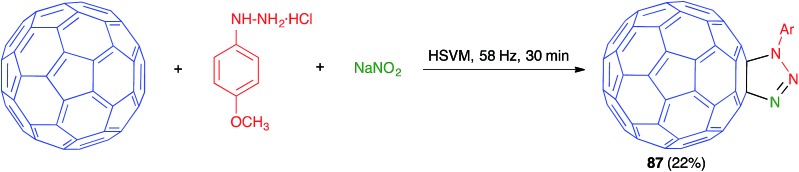
A three-component mechanochemical click reaction from fullerene.

As shown in Scheme 54, in another example of a mechanochemical three-component process based on a [3 + 2] dipolar cycloaddition on a fullerene substrate, Wang has reported the preparation of fullerotriazoline **87** by high-speed vibration milling treatment of C_60_ with an azide, generated *in situ* from the suitable phenylhydrazine and sodium nitrite.^75^

### 7.2. Macrocyclic nanostructures

**Scheme 55:**
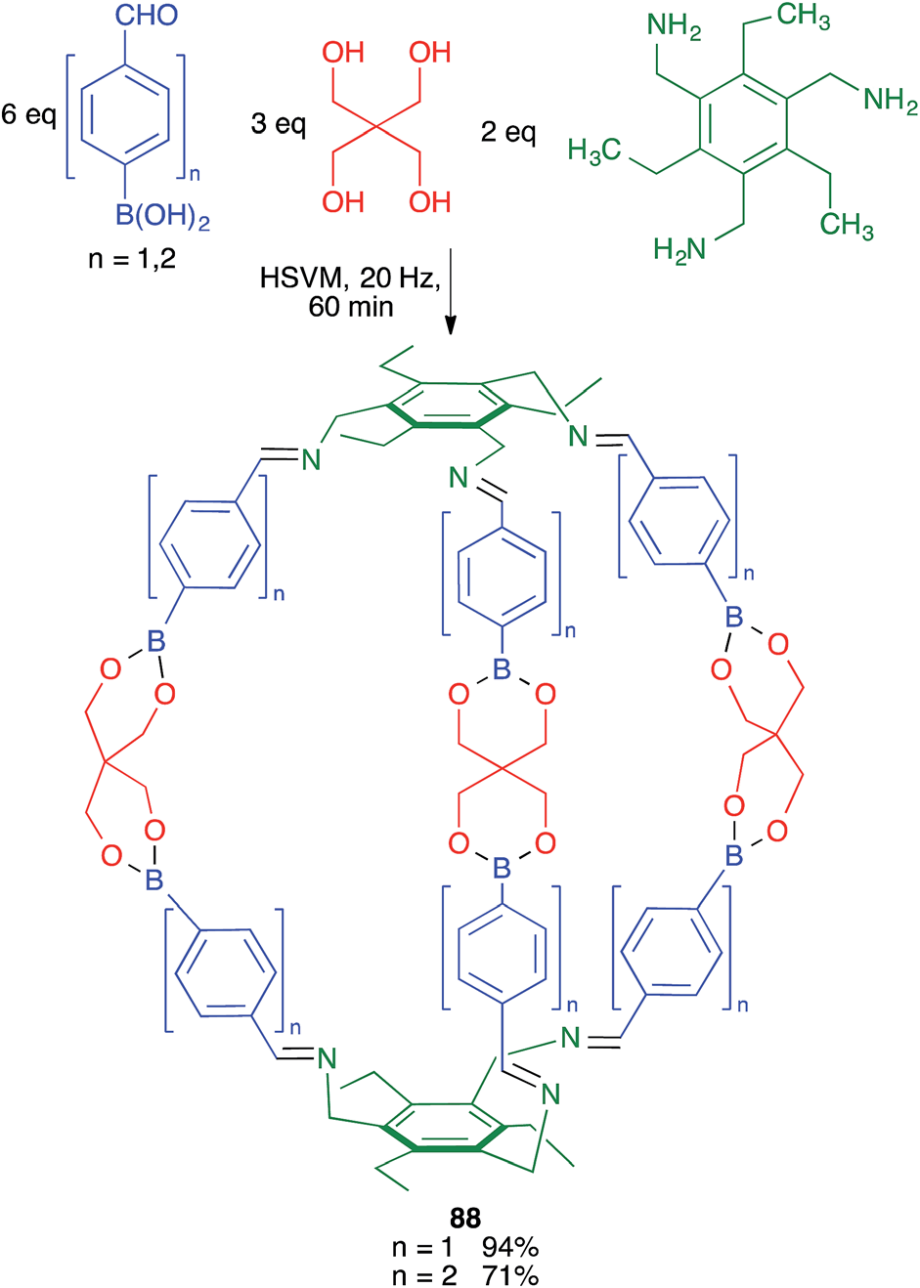
Mechanochemical synthesis of macromolecular cages.

Severin and coworkers prepared one of the first molecularly defined cage nanostructures (compounds **88**) from 4-formylbenzeneboronic acid, pentaerythritol and a triamine under high-speed vibration milling conditions, which gave better yields and purer products than solution techniques (Scheme 55).^76^

**Scheme 56:**
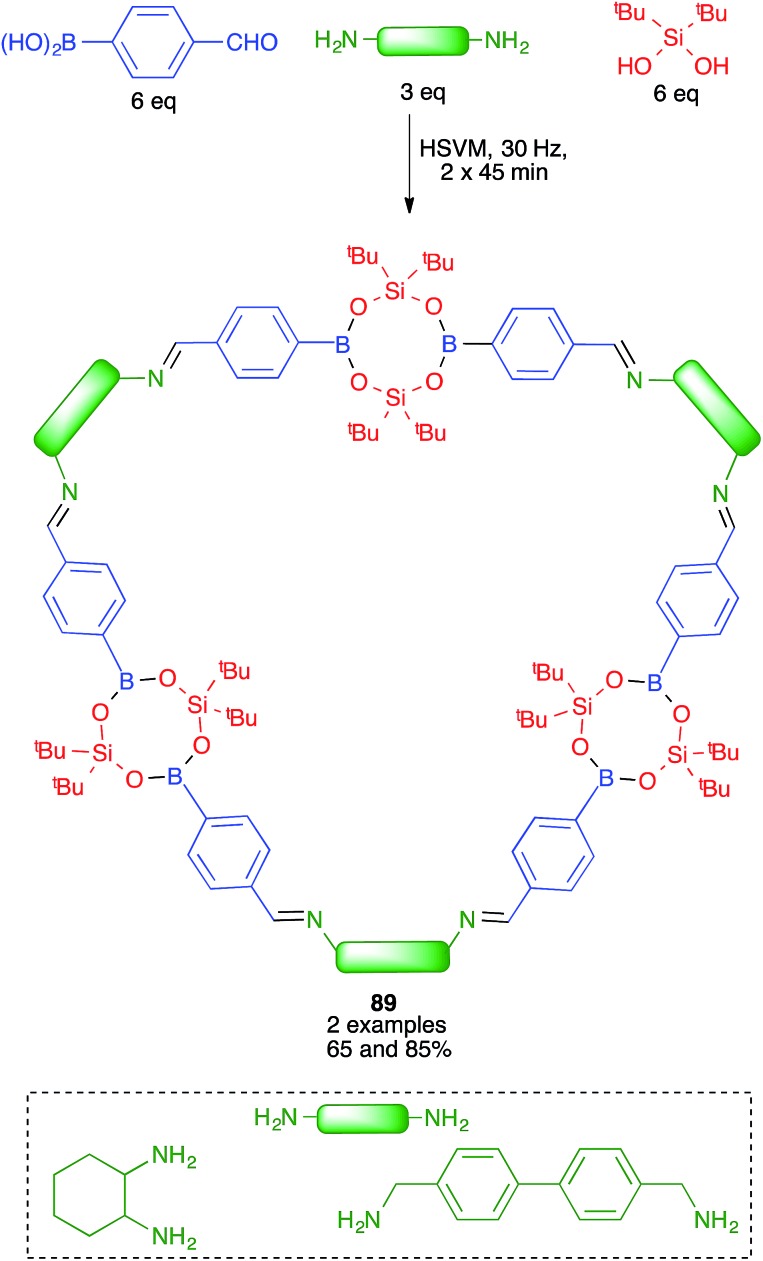
Mechanochemical synthesis of borasiloxane macrocycles.

The same group discovered later that borasiloxane-based macrocycles (compounds **89**) were readily available *via* multicomponent reactions from the same boronic acid, di(*tert*-butyl)silanediol and diamines (Scheme 56). As in the previous case, solution and high-speed vibration milling methodologies were compared for the preparation of these complex nanostructures, with the latter method proving advantageous in terms of yield.^77^

### 7.3. Metal–organic frameworks

Metal–organic frameworks (MOFs) are a subclass of coordination polymers that have interesting properties as functional materials and also have pharmaceutical applications (BioMOFs), for instance as drug carriers and as contrast agents in NMR imaging. Since the preparation of MOFs often requires the use of at least one starting material with poor solubility, it is an area in which mechanochemistry can play an important role.^78^

**Scheme 57:**
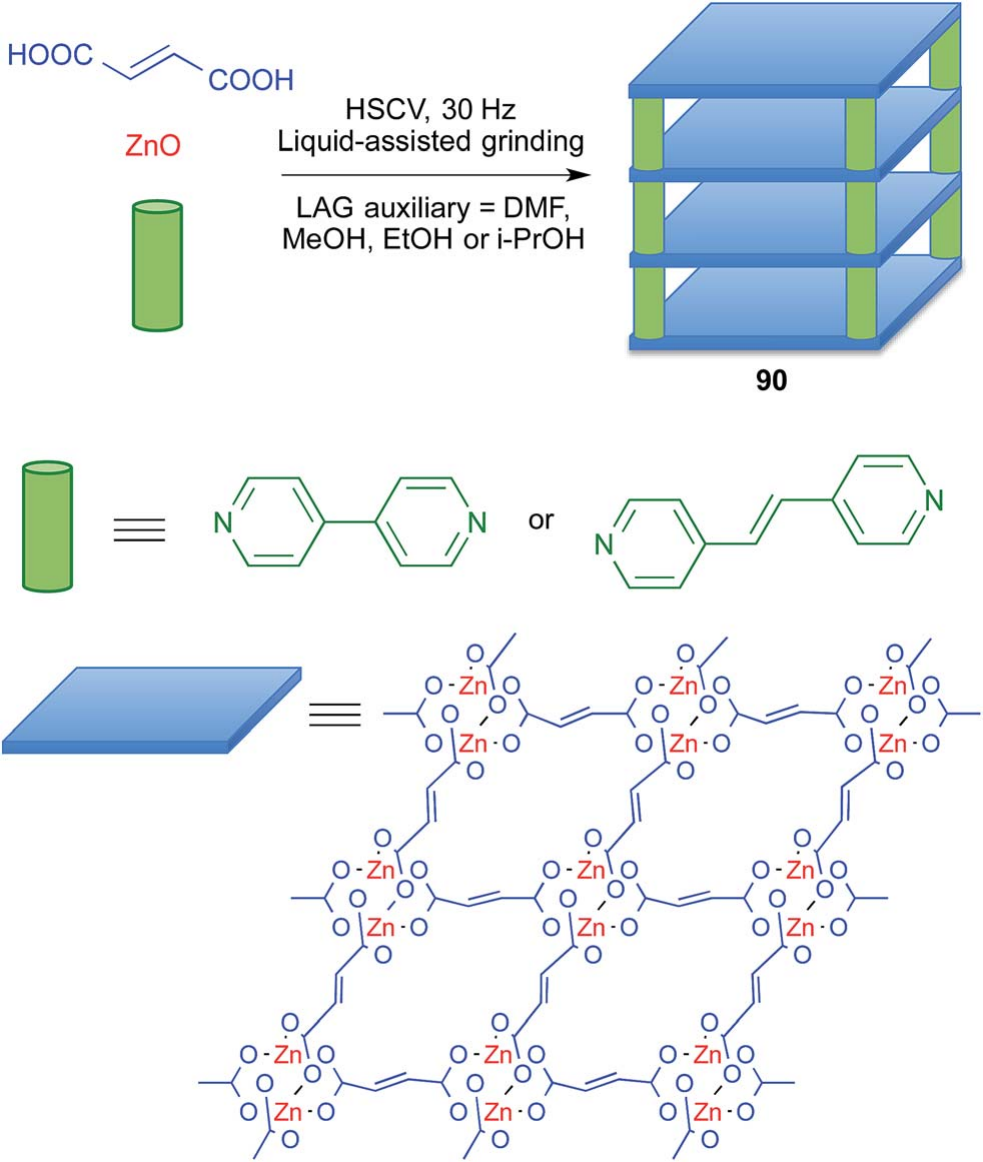
Mechanochemical synthesis of metal–organic frameworks.

Friščić and coworkers showed that liquid-assisted grinding of ZnO and fumaric acid afforded coordination polymers, and that addition of a third component such as 4,4′-bipyridyl or *trans*-1,2-di(4-pyridyl)ethylene furnished pillared MOFs **90** that behave as porous materials and are potentially useful for drug delivery purposes. They compared several liquids as grinding assistants, finding that dimethylformamide, methanol, ethanol and 2-propanol gave similar results, although the alcohols were preferred because they can be considered as more environmentally friendly (Scheme 57).^79^

The same group later proved that catalytic amount of some salts accelerated the reaction through templating effects, and used these improved conditions to prepare a related MOF from zinc oxide, terephthalic acid and 1,4-diazabicyclo[2.2.2]octane, DABCO. This was the first example of anion templating in mechanochemical synthesis.^80^ In all these experiments, thermal effects were discarded by running the mechanochemical reactions under a stream of air.

Isoreticular metal–organic frameworks (IRMOFs) have also been recently prepared by a multicomponent mechanochemical method.^81^

## 8. A summary of the potential advantages of the mechanochemical activation of multicomponent reactions

Using examples taken from previous Sections, we will summarize here the main potential advantages associated to the use of mechanochemical activation to promote multicomponent reactions.

### 8.1. Reduced use of organic solvents

In most cases, mechanochemical approaches avoid the use of significant amounts of solvents in the reaction media. More importantly in terms of waste generation, some mechanochemical reactions give analytically pure materials and therefore avoid the need for workup and purification procedures. Some examples include salen complexes **66** (ref. 67) and metal–organic frameworks **90**.^79^ In other cases, the reaction product is sufficiently pure to allow its final purification to analytical standards by recrystallization or washing, avoiding the need for chromatography, as in the case of borasiloxane-based macrocycles **89**.^77^

### 8.2. Telescoping of reactions

In some cases, the use of solvent-free mechanochemical conditions has allowed to telescope a multicomponent reaction with an additional step required for the synthesis of one of the starting materials in a way that was not possible in conventional conditions. Thus, the generalized Hantzsch pyrrole synthesis summarized in Schemes 14 and 15 and the accompanying discussion required the previous preparation of a α-iodoketone when carried out in solution, but this step could be incorporated into the sequential multicomponent process under mechanochemical conditions.^28^,^29^

### 8.3. Improved yields

**Scheme 58:**
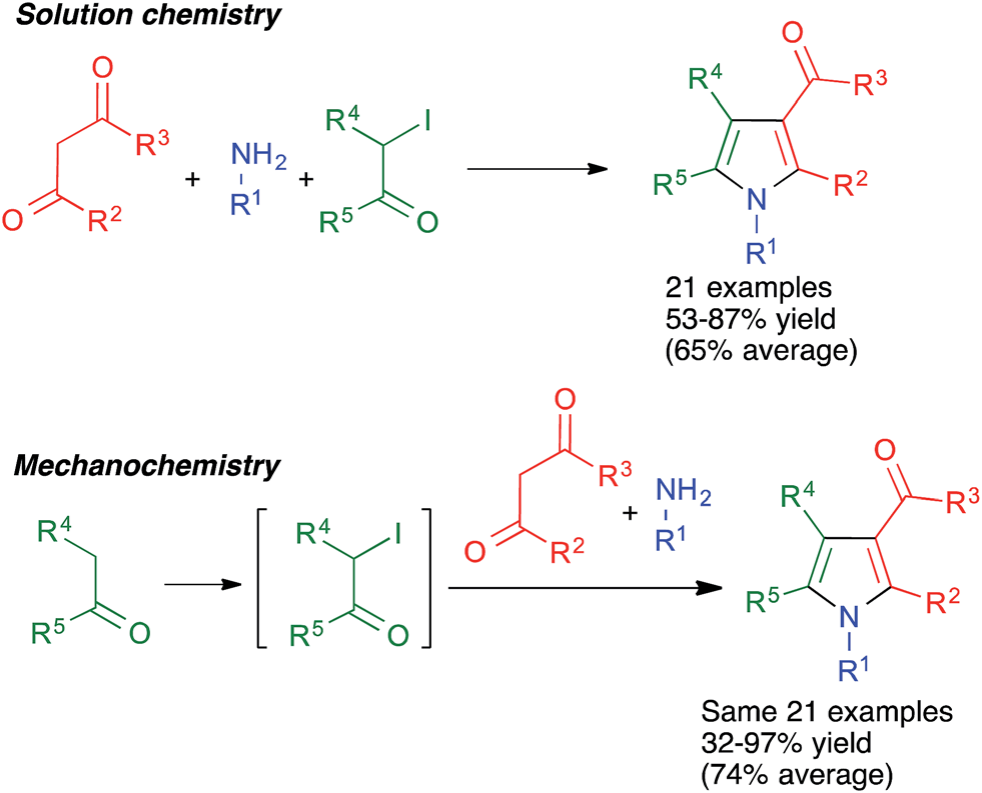
An example of a systematic comparison between the yields obtained for a multicomponent reaction in solution and in mechanochemical conditions.

Unfortunately, the literature hardly contains any systematic comparisons between the yields of multicomponent reactions when performed under mechanochemical and conventional conditions. One example of such a comparison was carried out for the above-mentioned generalized Hantzsch pyrrole synthesis. As shown in Scheme 58, the mechanochemical method gave significantly better yields in most cases in spite of comprising an additional step. The only exception were the reactions starting from acetone, which under mechanochemical conditions is probably in equilibrium between the liquid and the vapor phase, and in the latter collisions with the ball are not effective.^29^ Another example is that of cage nanostructures **88**, which were obtained in 56% yield in solution *vs.* 94% in mechanochemical conditions for *n* = 1 and <40% (with low purity) *vs.* 71% for *n* = 2.^76^

### 8.4. Reduced reaction times

One striking example comes from the solvent-free, mechanochemical synthesis of BODIPY dyes described in Scheme 45 and the accompanying discussion. This reaction had the advantage of requiring very short reaction times (5 min) when compared to conventional solution chemistry, which required from several hours to days to reach the same final products.^66^

### 8.5. Improved selectivity

As summarized in Scheme 37 and the accompanying discussion, the mechanochemical Povarov reaction between *in situ*-generated *N*-aryl aldimines and styrene gave the corresponding *cis*-2,4-diphenyl-1,2,3,4-tetrahydroquinolines **51** with full diastereoselectivity, which represented an improvement over the reaction in solution. This result was explained by the increased preference for the formation of the kinetic product due to the high concentration of the reactants.^58^

### 8.6. Use of insoluble starting materials

Compounds that show low solubilities in common organic solvents and also in water are often attractive substrates for modification *via* multicomponent transformations. In these cases, solvent-free approaches are mandatory and therefore mechanochemistry can be useful. As mentioned in Section 7.1, fullerenes, carbon nanotubes and graphite are examples of such barely handleable substrates. For the same reason, the multicomponent construction of materials such as hybrid perovskites benefits greatly from mechanochemical activation.^82^

### 8.7. Improved safety

Ball milling is normally performed in sealed steel containers, and can therefore be viewed as safer than chemistry carried out in glassware when potentially explosive compounds such as azides are handled or generated in the course of a reaction. One example is the copper(i)-catalyzed azide alkyne cycloaddition CuAAC reaction leading to **42**.^45^

### 8.8. New types of reactivity

In some cases, new multicomponent transformations can be achieved under mechanochemical conditions that are not possible in solution. For instance, the reaction between *in situ*-generated β-enaminones and chalcones in the presence of AlCl_3_ afforded 1,4,6-triaryl-1,4-dihydropyridine derivatives **47** (Scheme 33) under mechanochemical conditions, but gave carbocycles in solution.^51^ The preparation of cyclopalladated complexes **72** constitutes another example of this kind of situations, since the last step could not be carried out in solution.^69^

## 9. Conclusions

Mechanochemical synthesis, based on the direct absorption of mechanical energy to induce chemical transformations, has come a long way towards becoming a mainstream tool in synthetic laboratories. However, its combination with multiple bond-forming reactions as a pathway towards generating synergy in the reduction of the number of isolation and purification steps in synthetic operations has received relatively little attention. Besides their role in promoting sustainable chemistry, mechanochemical multicomponent processes lead to new opportunities in the discovery of new synthetically useful transformations. Mechanochemical processes are normally performed under rather unique conditions involving very high reagent concentrations and in the absence of solvation effects, which sometimes leads to alterations in product selectivity and may therefore enable new modes of reactivity. Furthermore, the fact that mechanochemical conditions allow the use of solid starting materials permits designing new multicomponent reactions that are not viable in conventional solution conditions. Many additional advantages, including the possibility to telescope reactions, improved yields, reduced reaction times, improved selectivities and improved safety have been described for mechanochemical multicomponent reactions.

In this context, we hope that this Perspective article, by critically summarizing the progress made so far in the area of mechanochemical multicomponent chemistry, will stimulate researchers to take this promising pathway towards the design of new synthetic methods and the development of more efficient and sustainable chemical transformations.

## Conflicts of interest

There are no conflicts to declare.
